# Supplementary figures and images for: Impaired migration and lung invasion of human melanoma by a novel small molecule targeting the transmembrane domain of death receptor p75NTR
Source: EMBO Mol Med. 2025 Sep 17;17(10):2661–90. doi: 10.1038/s44321-025-00297-1 (PMC12514245; doi:10.1038/s44321-025-00297-1)

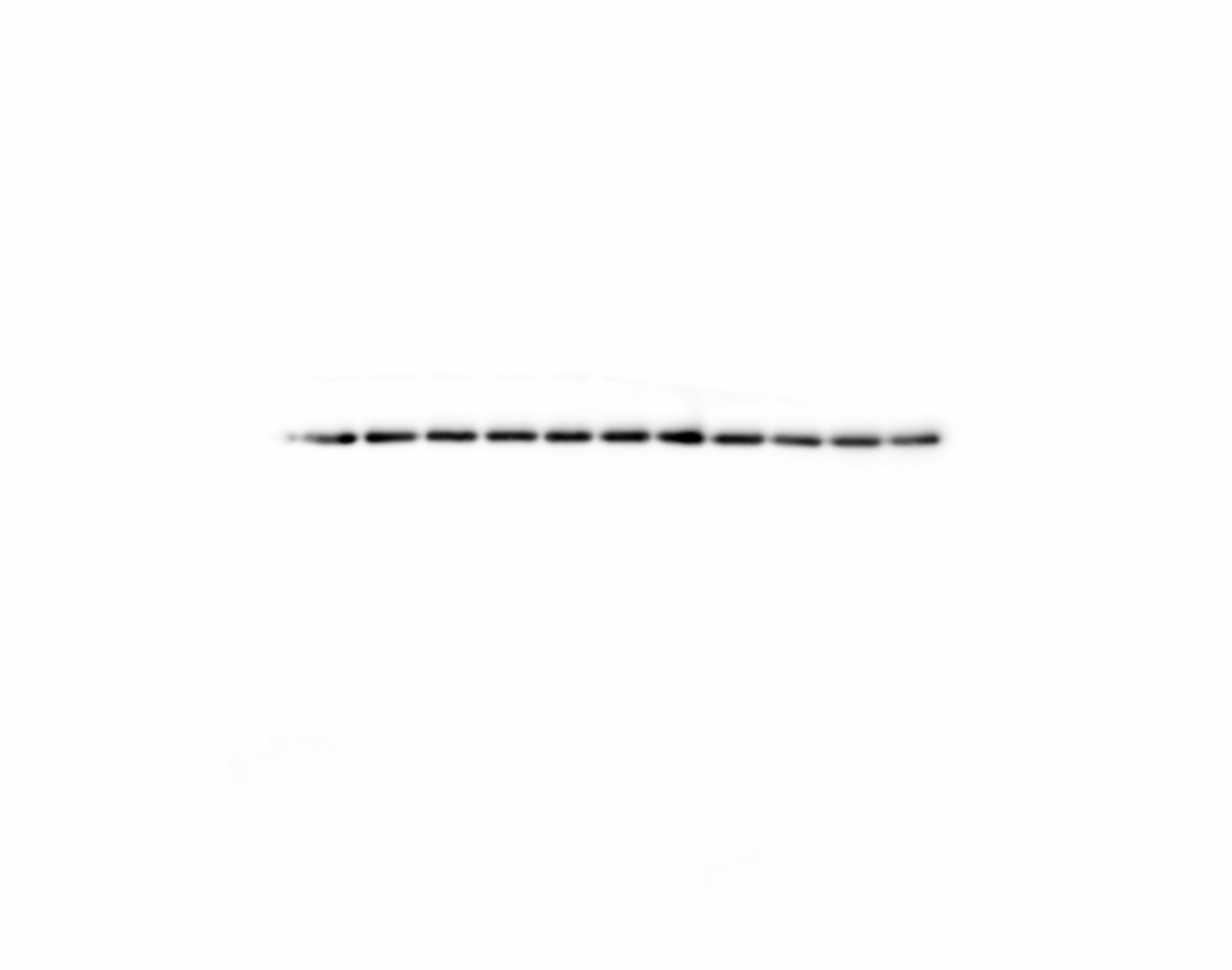

Supplement: Supplementary file 3 — Source data Fig. 2 [file 44321_2025_297_MOESM3_ESM.zip › Figure 2/new 2C GAPDH.tif]

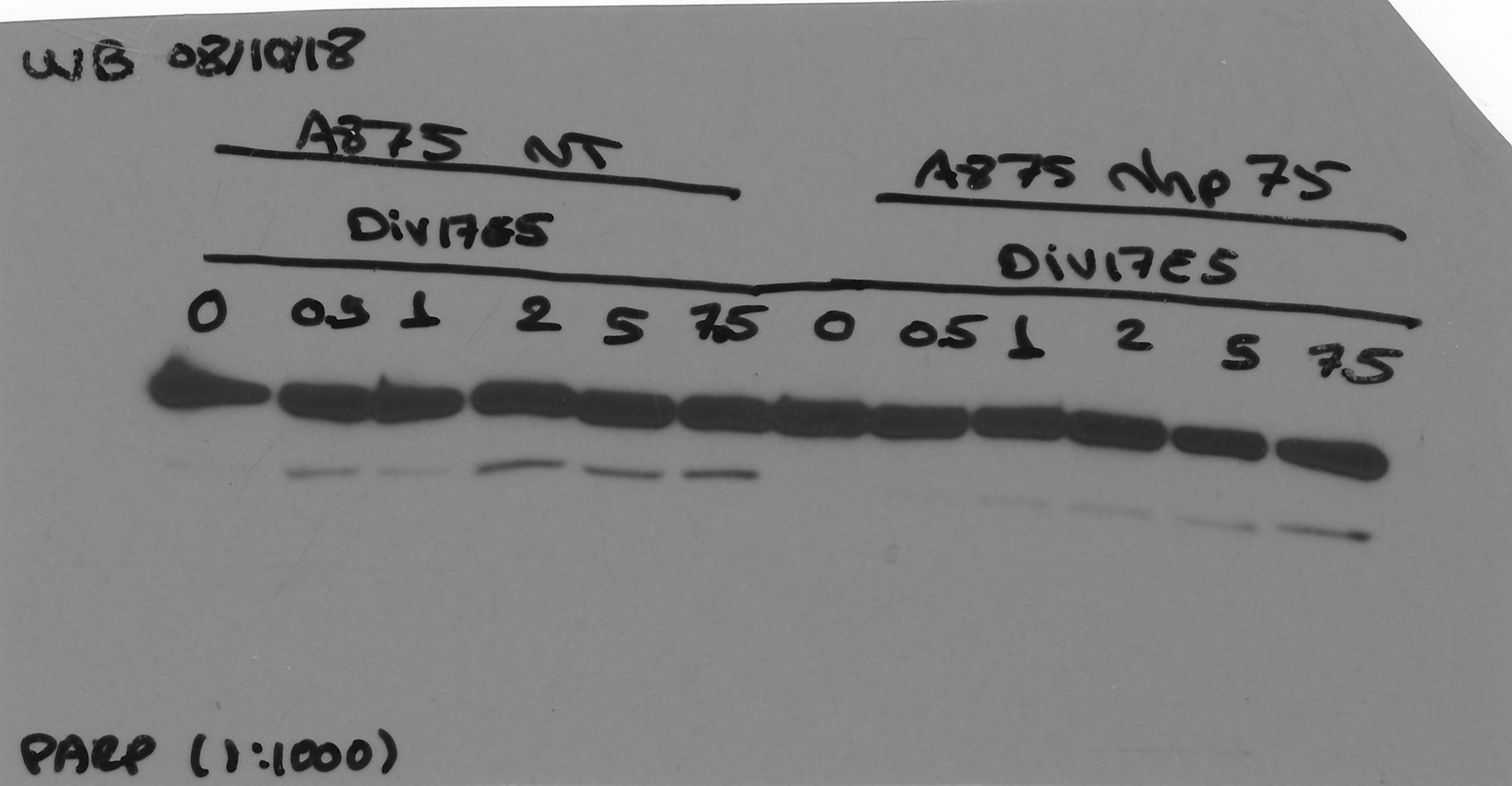

Supplement: Supplementary file 3 — Source data Fig. 2 [file 44321_2025_297_MOESM3_ESM.zip › Figure 2/Fig 2B PARP.tif]

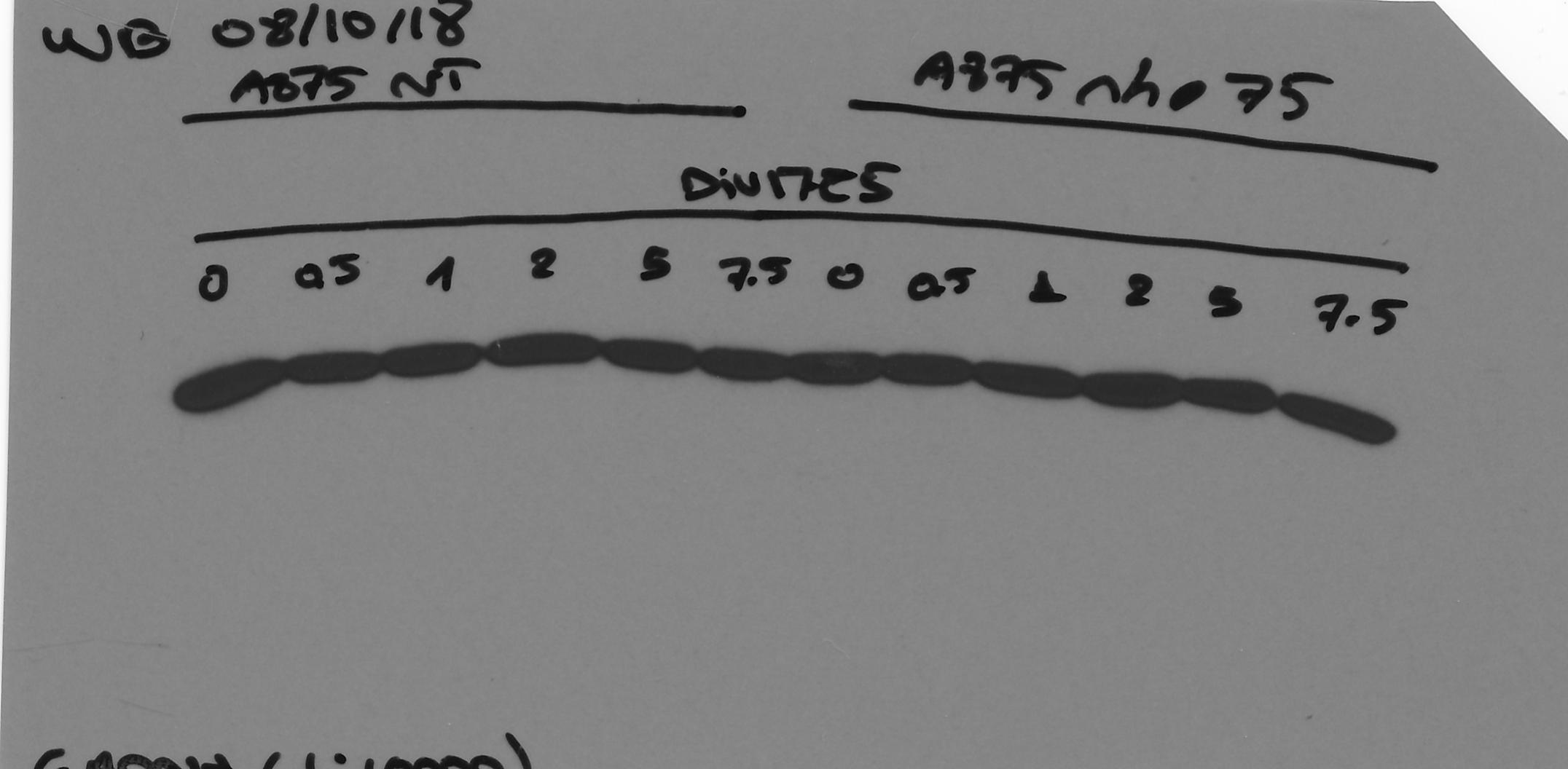

Supplement: Supplementary file 3 — Source data Fig. 2 [file 44321_2025_297_MOESM3_ESM.zip › Figure 2/Fig 2B GAPDH.tif]

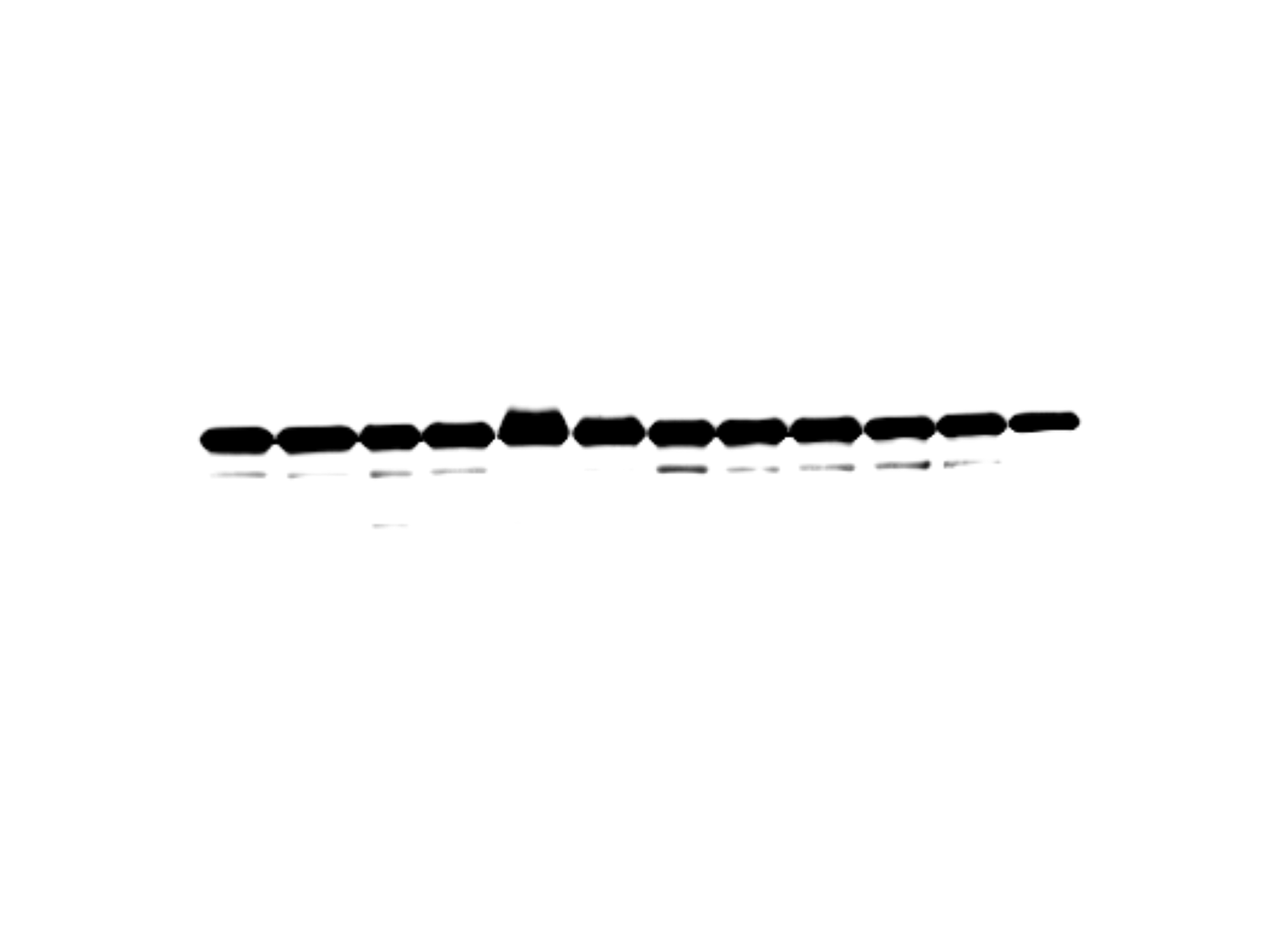

Supplement: Supplementary file 3 — Source data Fig. 2 [file 44321_2025_297_MOESM3_ESM.zip › Figure 2/new 2C PARP.tif]

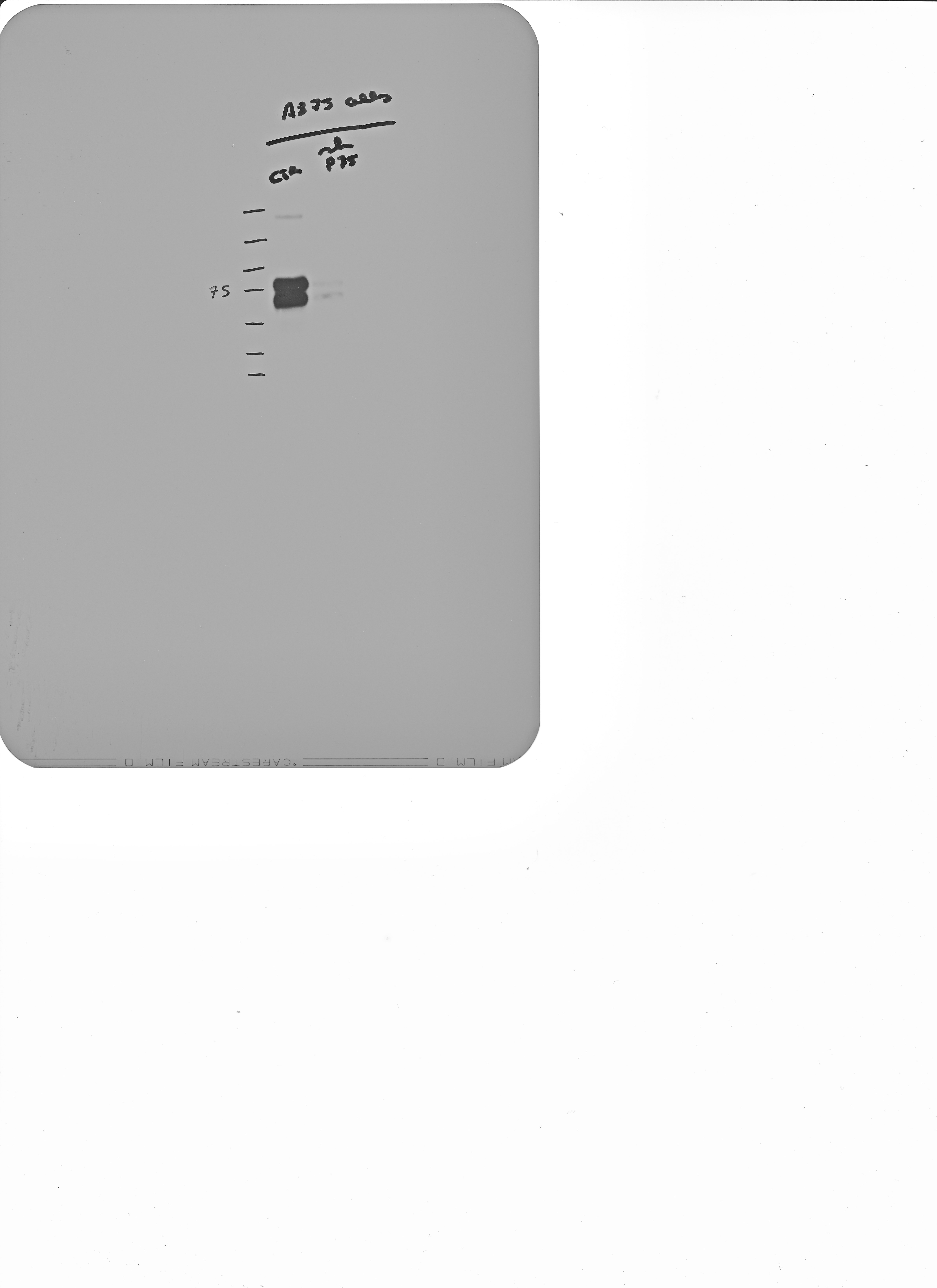

Supplement: Supplementary file 3 — Source data Fig. 2 [file 44321_2025_297_MOESM3_ESM.zip › Figure 2/Fig 2A p75.jpg]

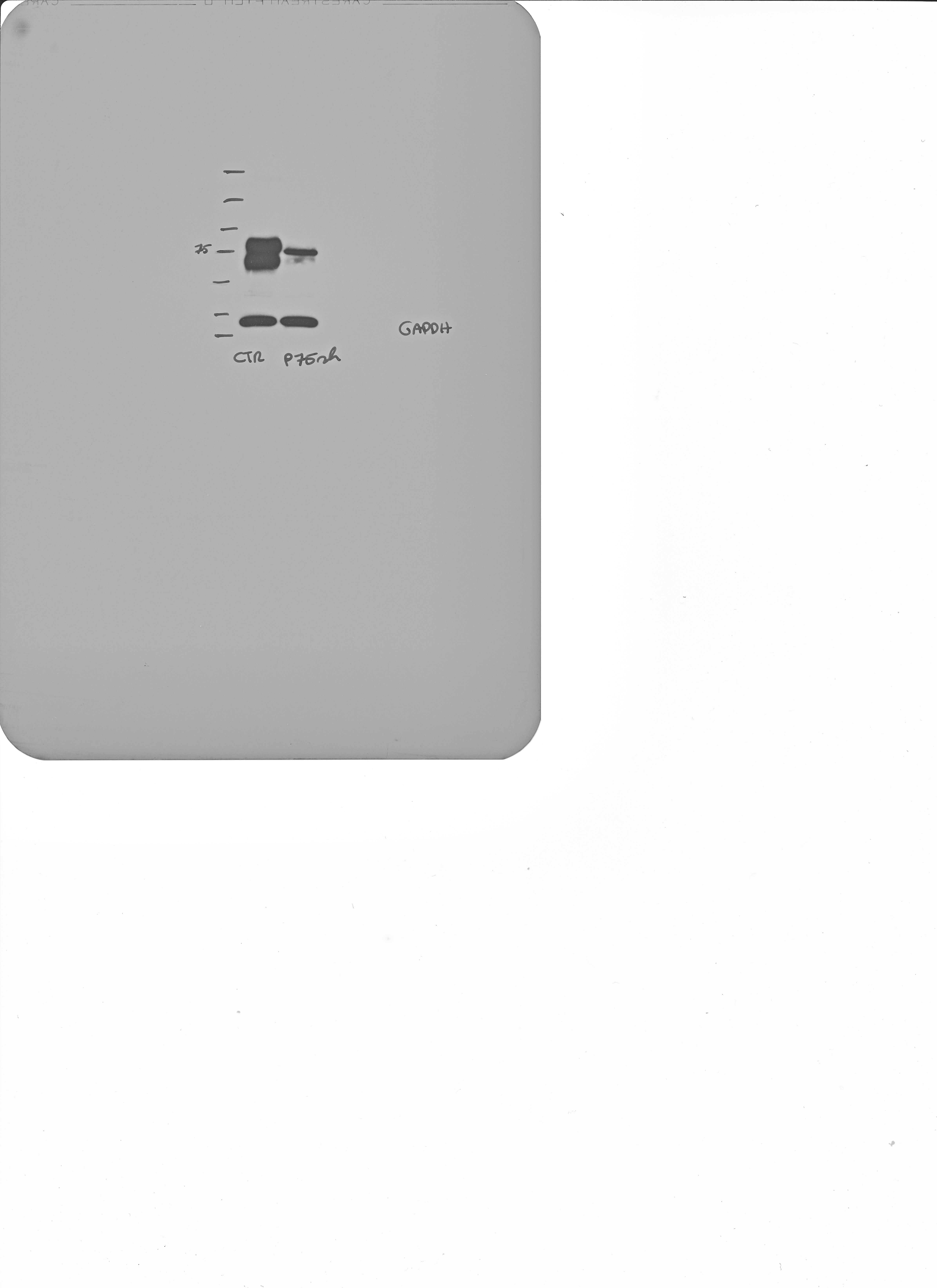

Supplement: Supplementary file 3 — Source data Fig. 2 [file 44321_2025_297_MOESM3_ESM.zip › Figure 2/Fig 2A GAPDH.jpg]

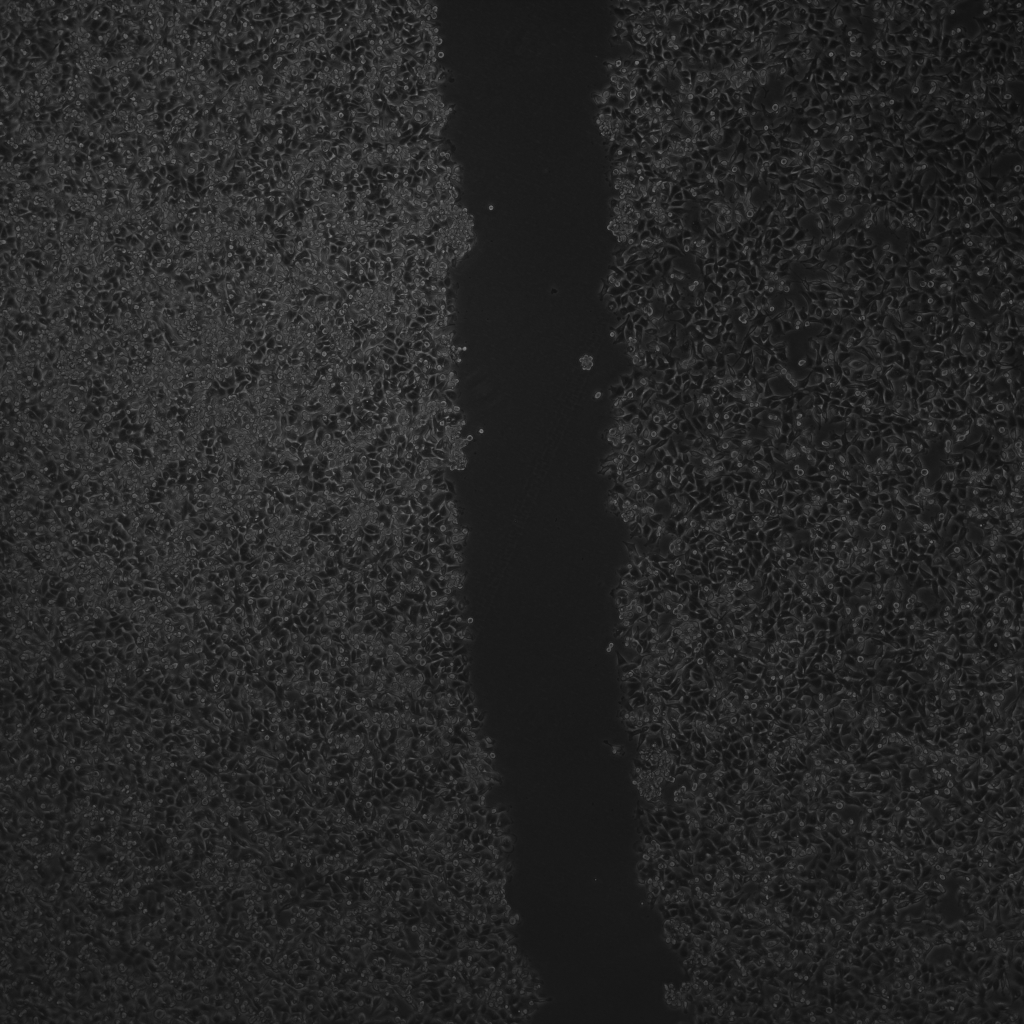

Supplement: Supplementary file 4 — Source data Fig. 3 [file 44321_2025_297_MOESM4_ESM.zip › Figure 3A/A875NT_DMSO_0h.TIF]

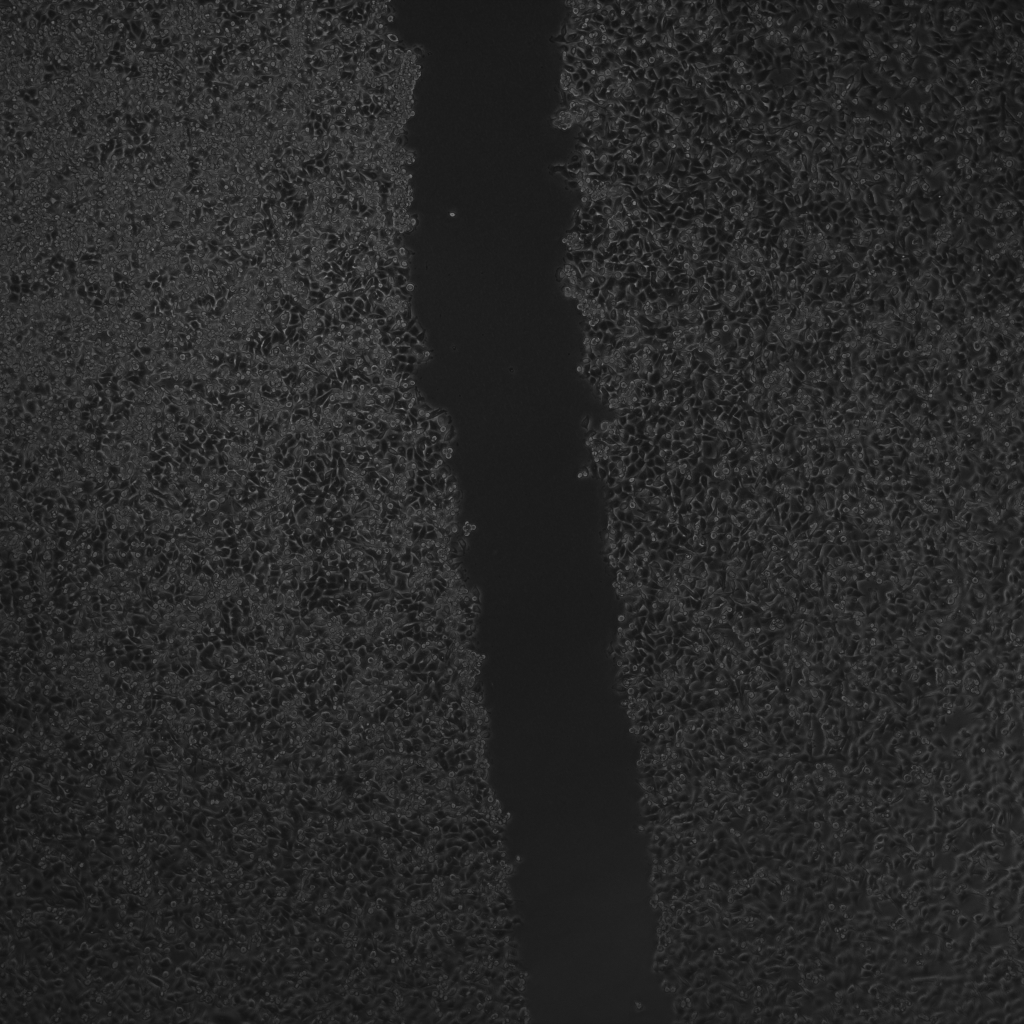

Supplement: Supplementary file 4 — Source data Fig. 3 [file 44321_2025_297_MOESM4_ESM.zip › Figure 3A/A875NT_5umQ_VD_0h.TIF]

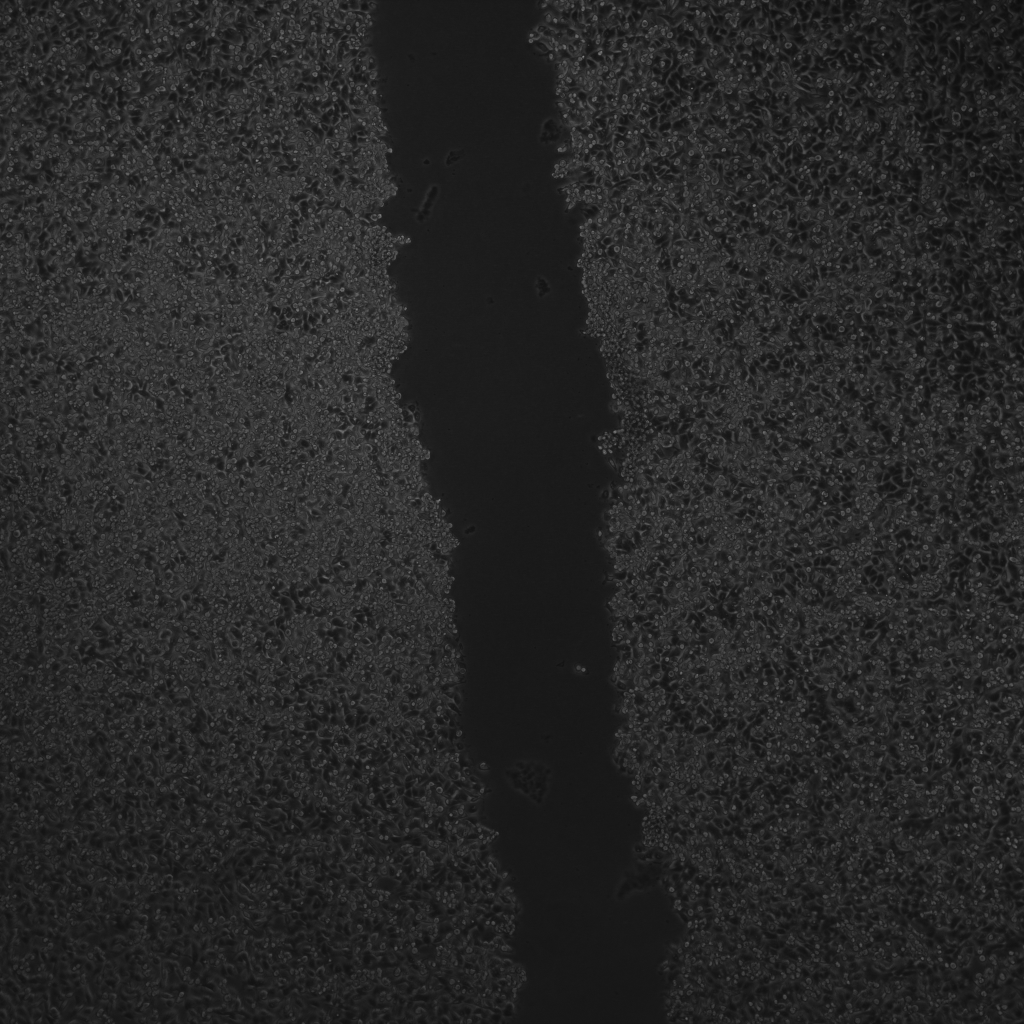

Supplement: Supplementary file 4 — Source data Fig. 3 [file 44321_2025_297_MOESM4_ESM.zip › Figure 3A/A875NT_10uMdiv17E5_0h.TIF]

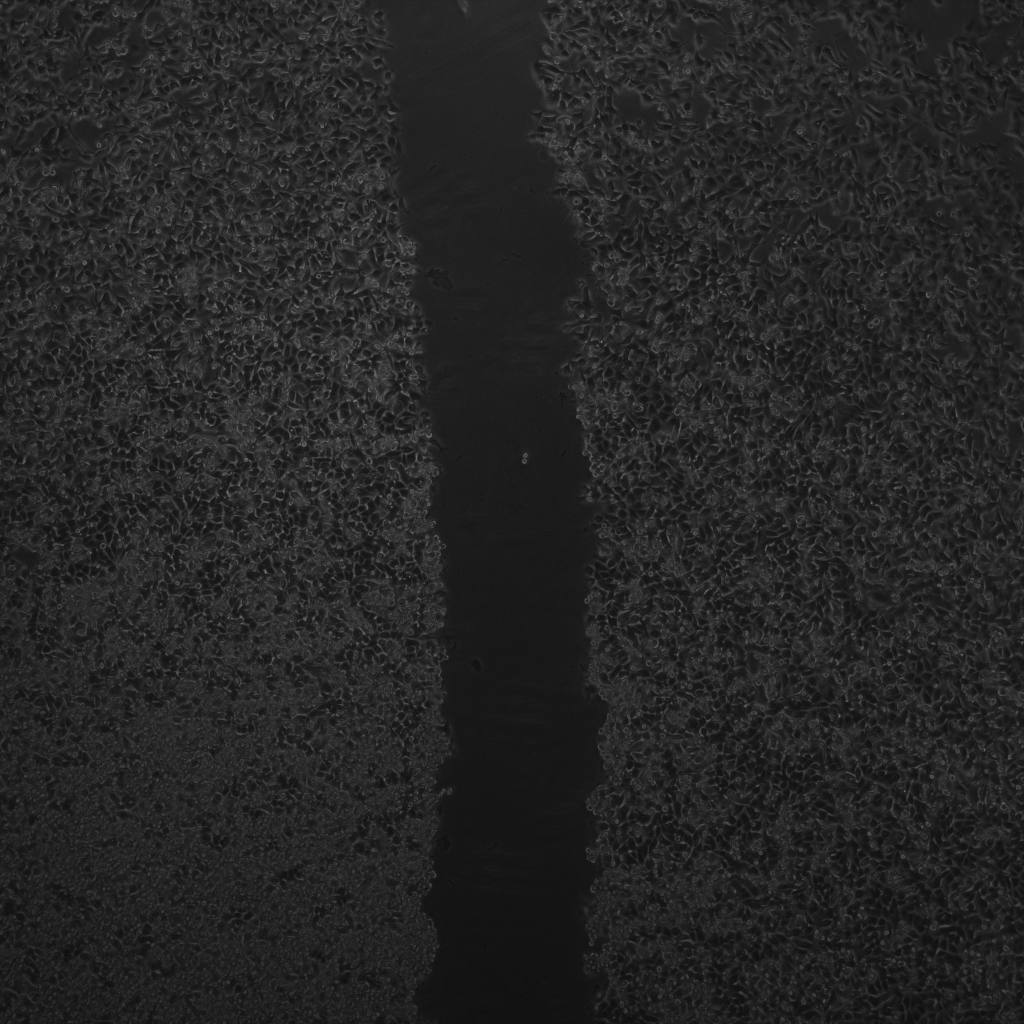

Supplement: Supplementary file 4 — Source data Fig. 3 [file 44321_2025_297_MOESM4_ESM.zip › Figure 3A/A875NT_5uM Q_VD_poH+10uMdiv17E5_0h.TIF]

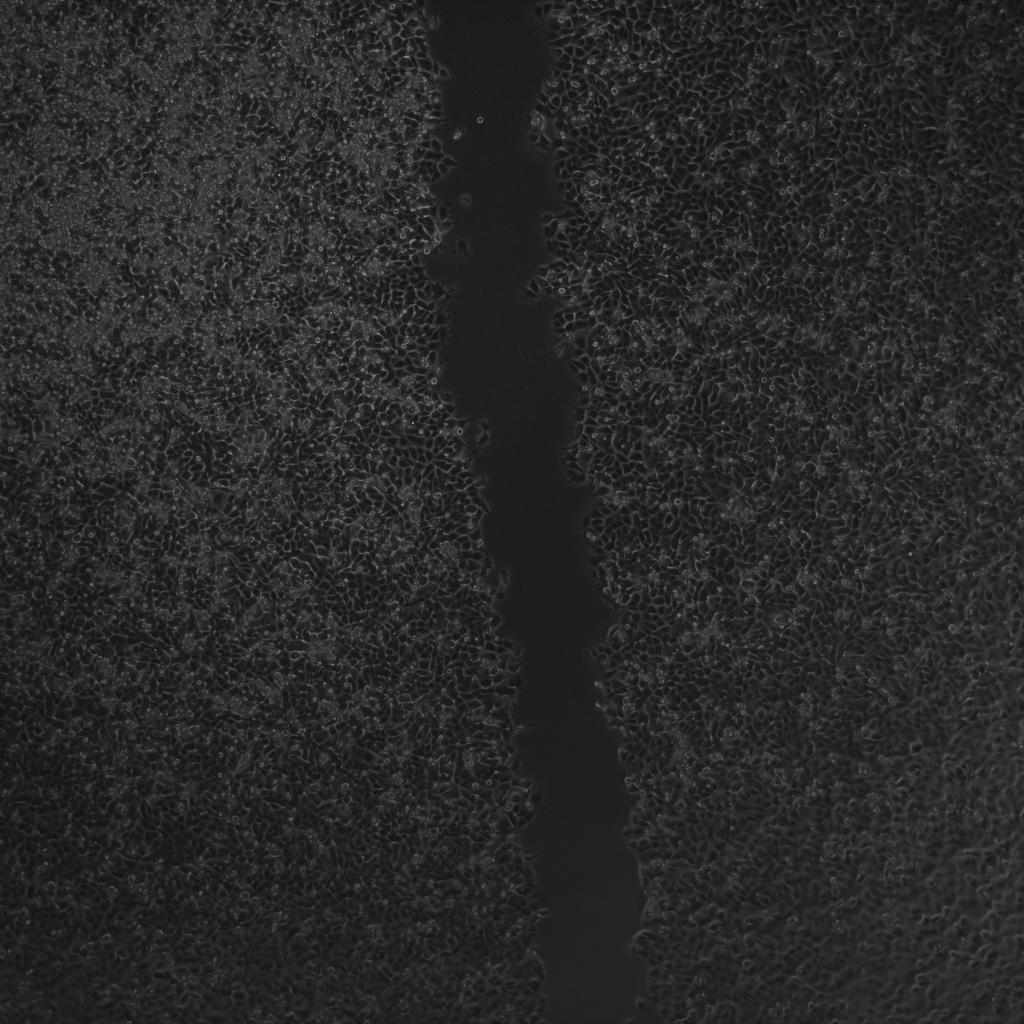

Supplement: Supplementary file 4 — Source data Fig. 3 [file 44321_2025_297_MOESM4_ESM.zip › Figure 3A/A875NT_5umQ_VD_12h.TIF]

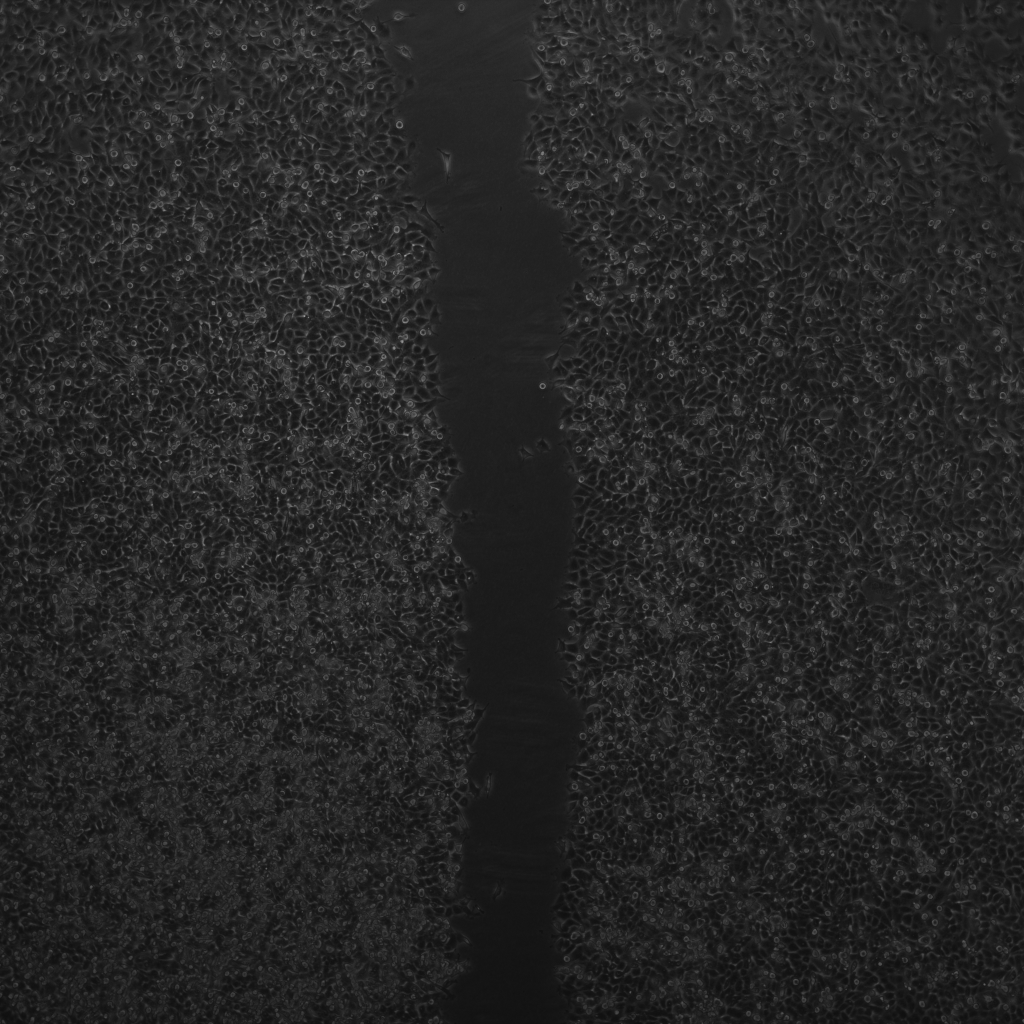

Supplement: Supplementary file 4 — Source data Fig. 3 [file 44321_2025_297_MOESM4_ESM.zip › Figure 3A/A875NT_5uM Q_VD_poH+10uMdiv17E5_12h.TIF]

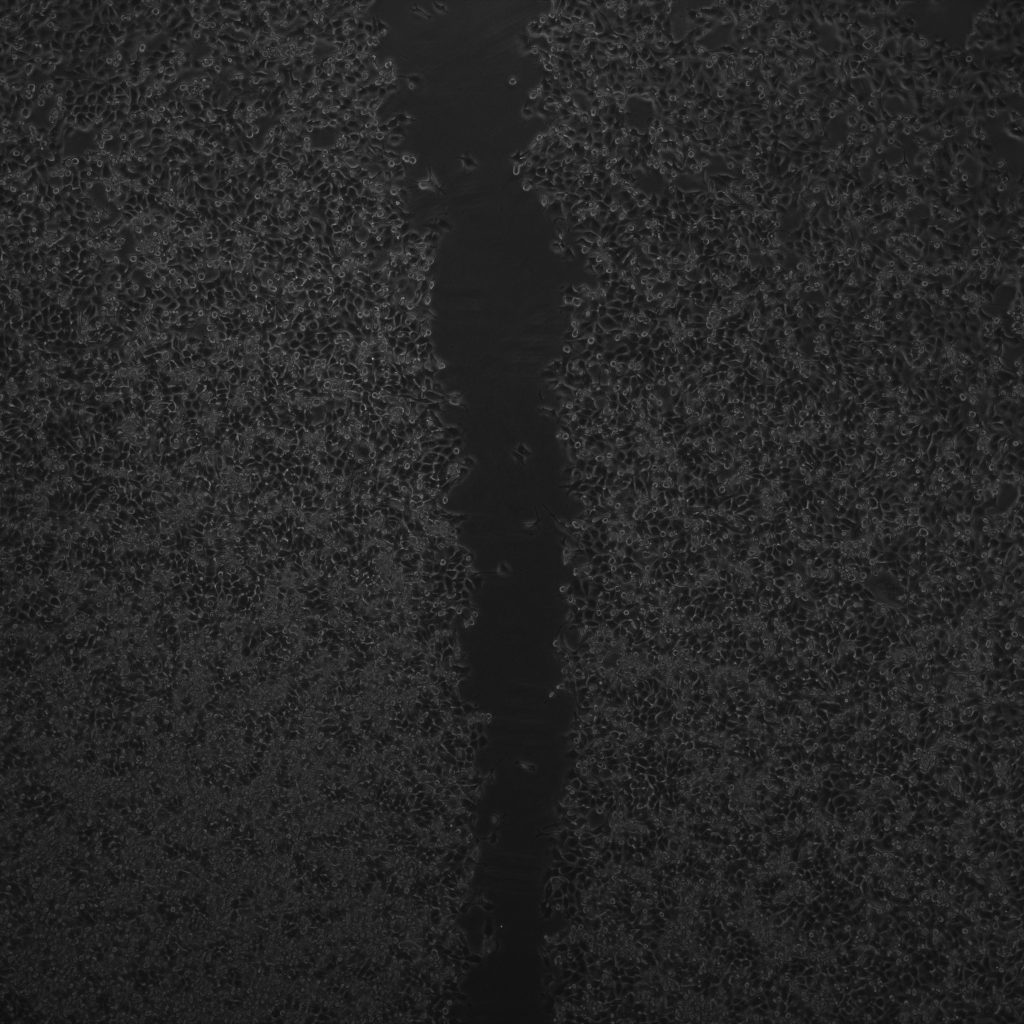

Supplement: Supplementary file 4 — Source data Fig. 3 [file 44321_2025_297_MOESM4_ESM.zip › Figure 3A/A875NT_5uM Q_VD_poH+10uMdiv17E5_24h.TIF]

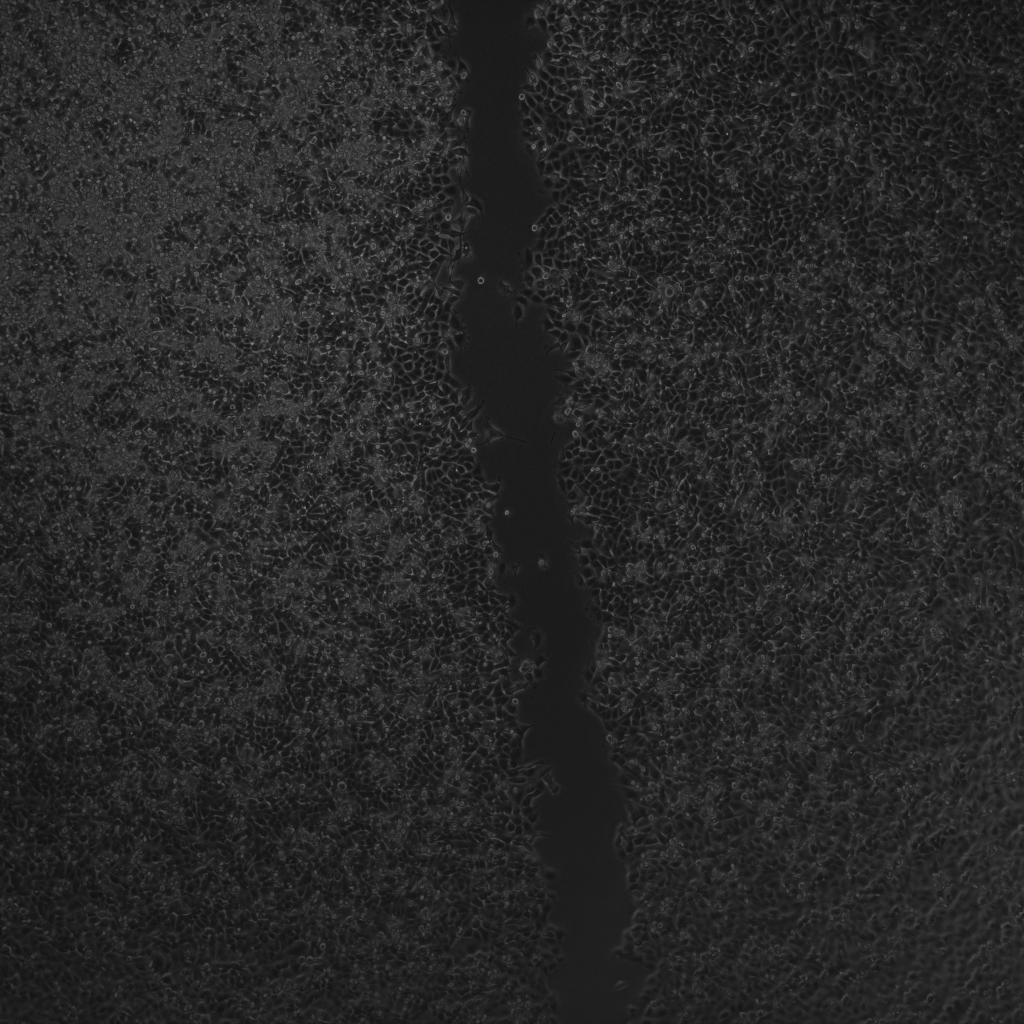

Supplement: Supplementary file 4 — Source data Fig. 3 [file 44321_2025_297_MOESM4_ESM.zip › Figure 3A/A875NT_5umQ_VD_24h.TIF]

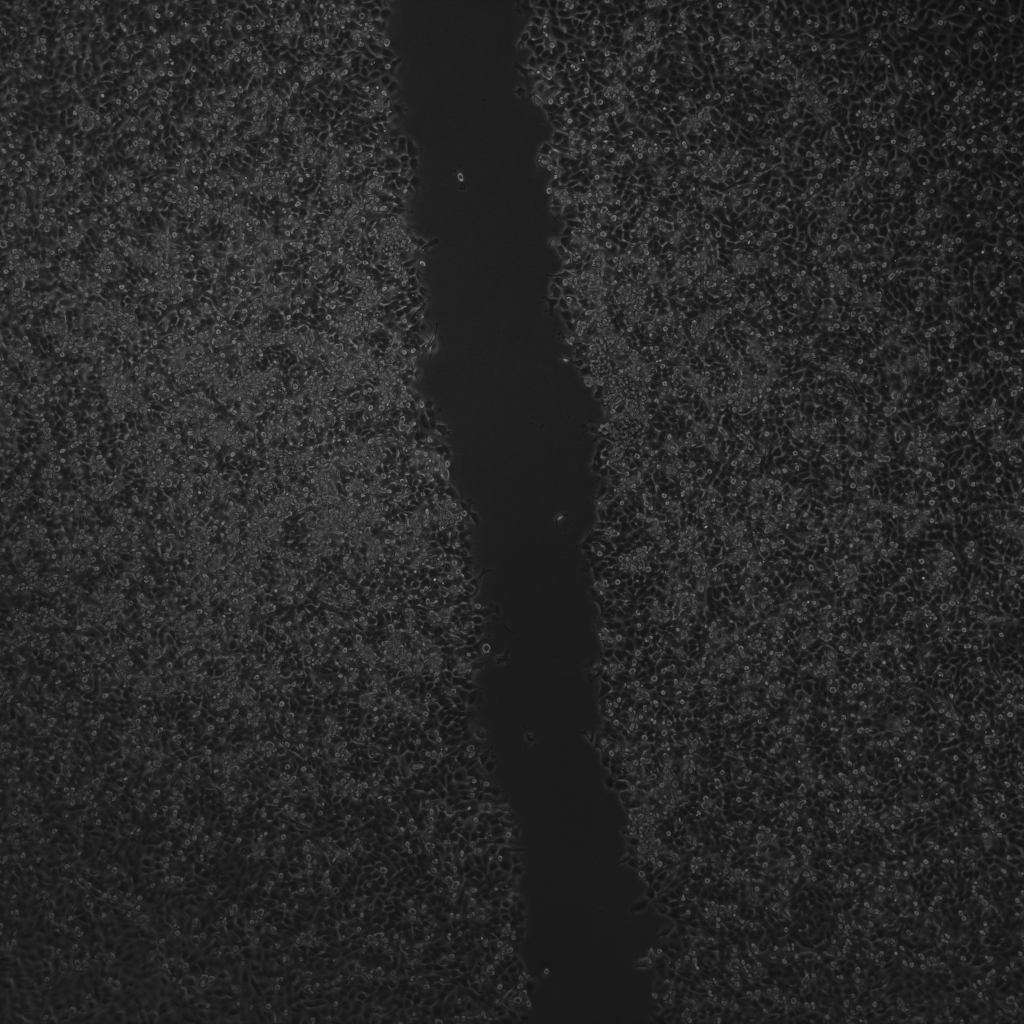

Supplement: Supplementary file 4 — Source data Fig. 3 [file 44321_2025_297_MOESM4_ESM.zip › Figure 3A/A875NT_10uMdiv17E5_12h.TIF]

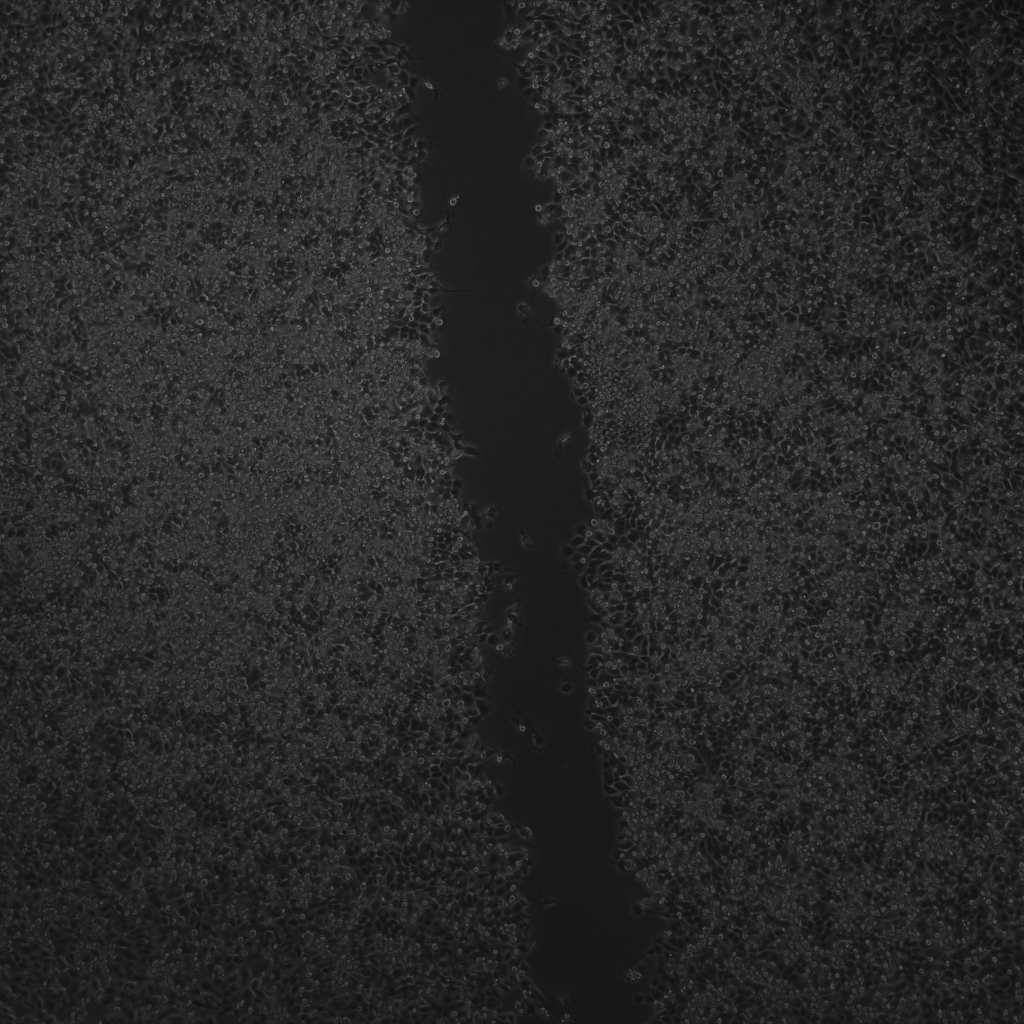

Supplement: Supplementary file 4 — Source data Fig. 3 [file 44321_2025_297_MOESM4_ESM.zip › Figure 3A/A875NT_10uMdiv17E5_24h.TIF]

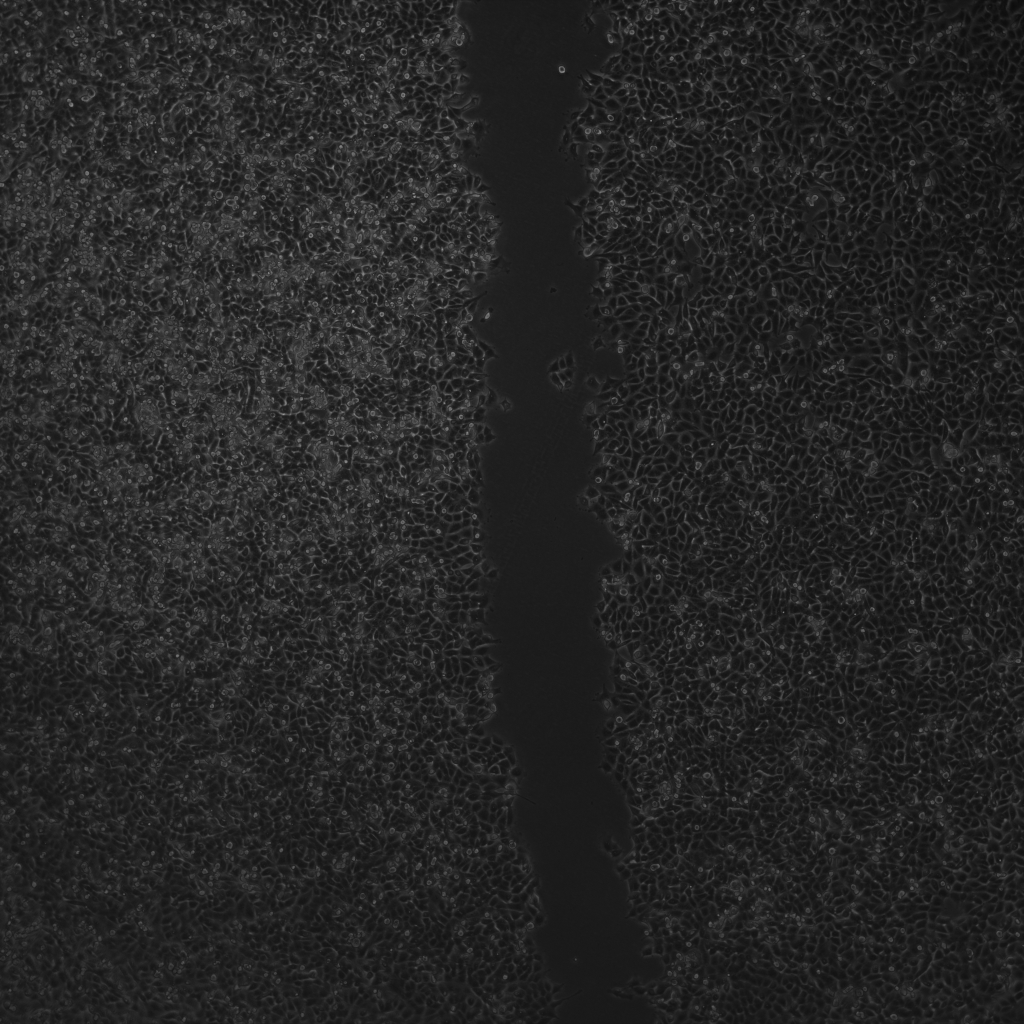

Supplement: Supplementary file 4 — Source data Fig. 3 [file 44321_2025_297_MOESM4_ESM.zip › Figure 3A/A875NT_DMSO_12h.TIF]

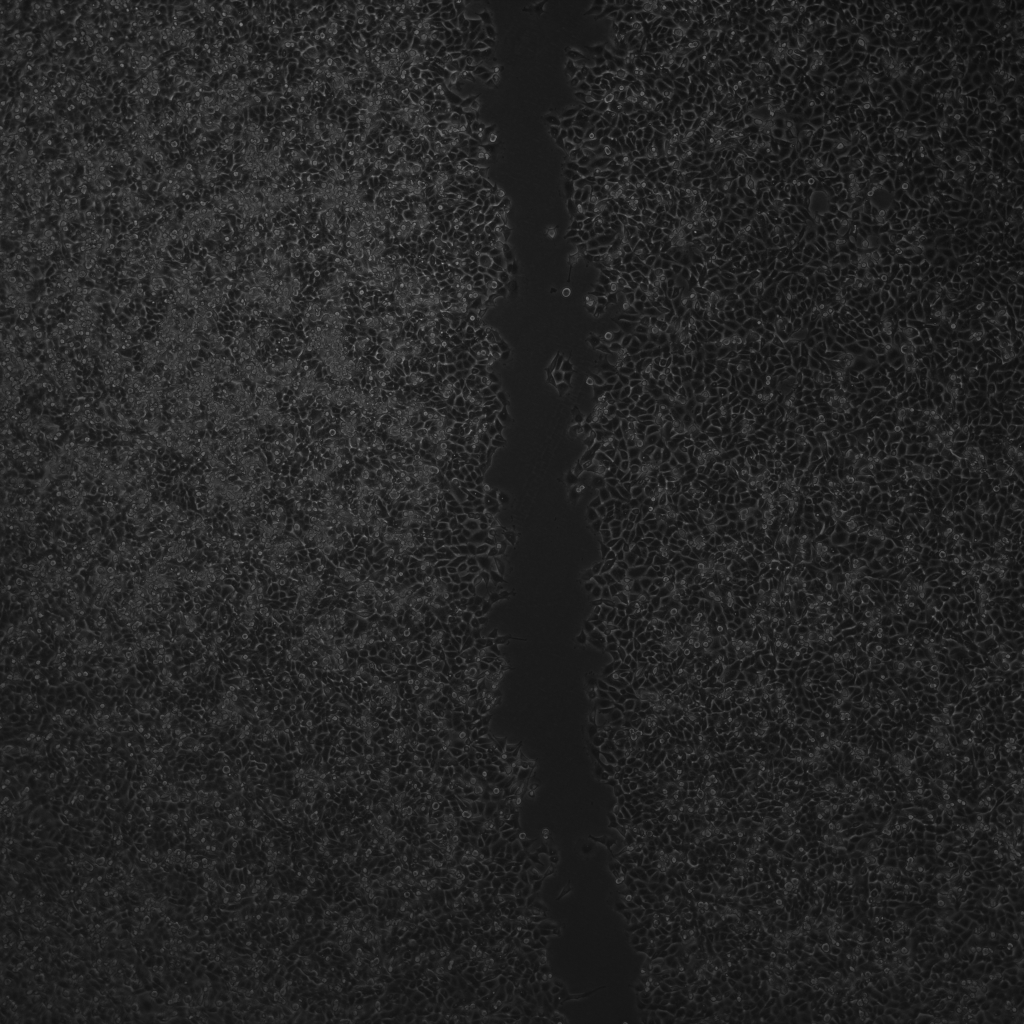

Supplement: Supplementary file 4 — Source data Fig. 3 [file 44321_2025_297_MOESM4_ESM.zip › Figure 3A/A875NT_DMSO_24h.TIF]

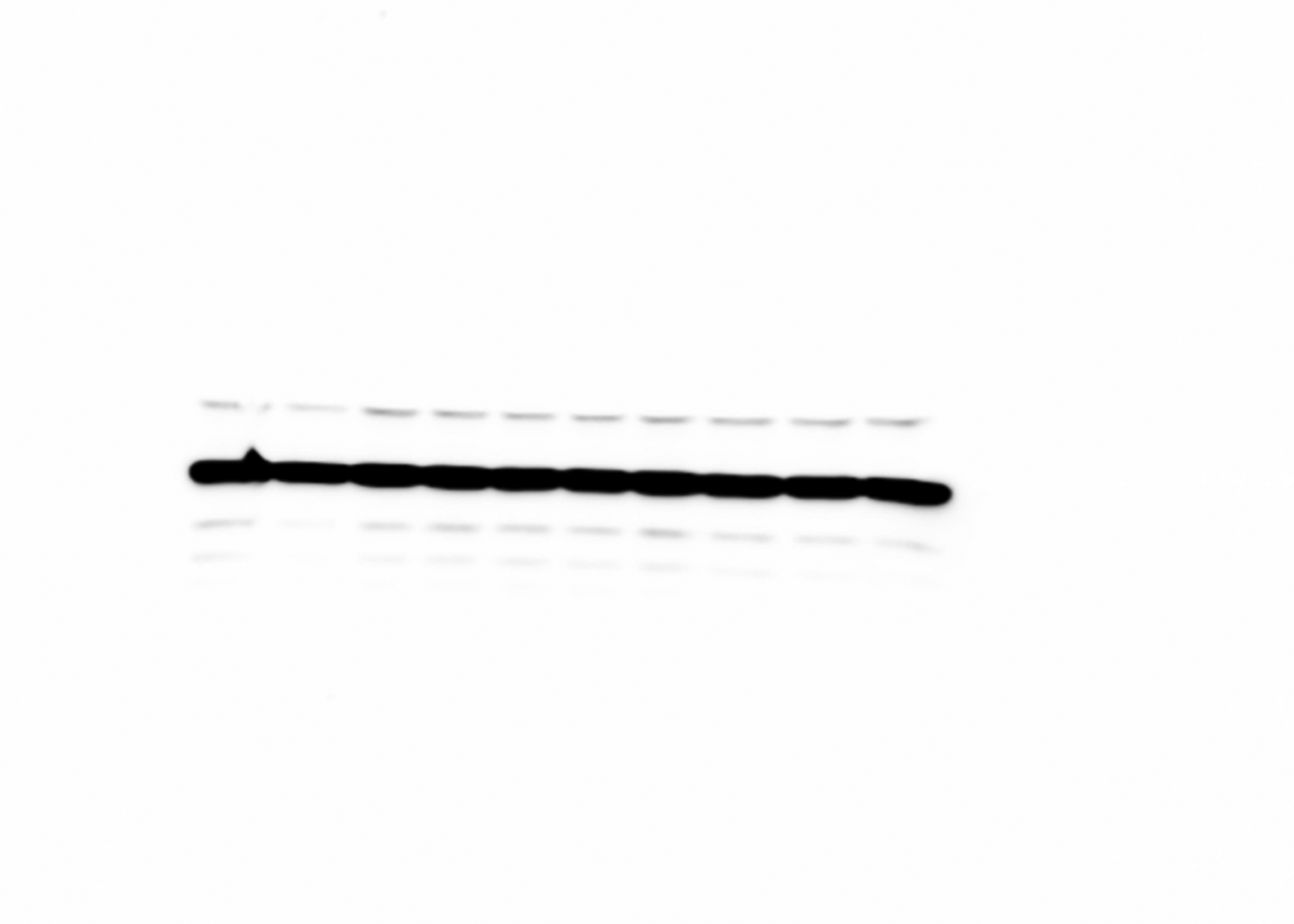

Supplement: Supplementary file 7 — Source data Fig. 6 [file 44321_2025_297_MOESM7_ESM.zip › Figure 6A/new 6A GAPDH.tif]

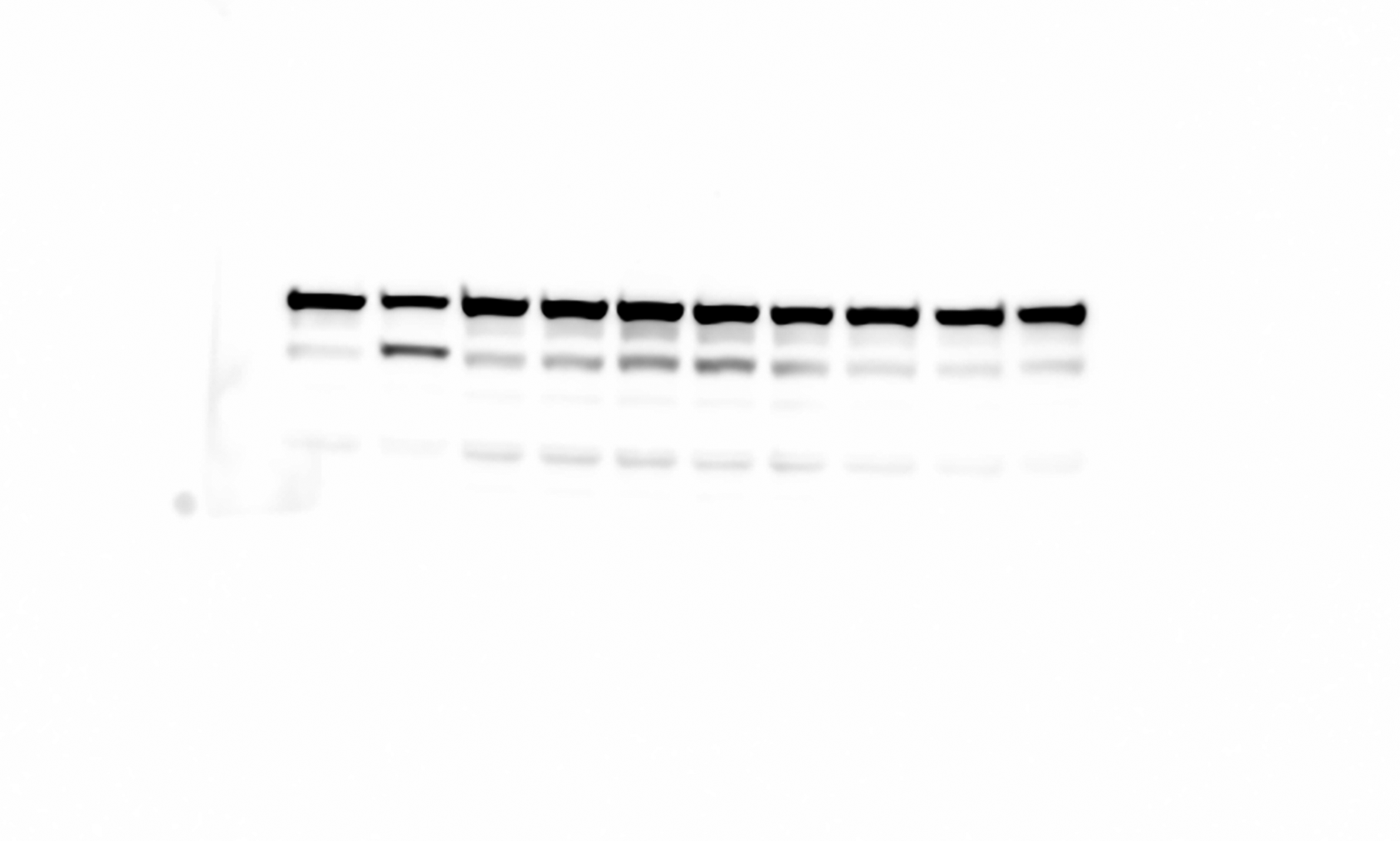

Supplement: Supplementary file 7 — Source data Fig. 6 [file 44321_2025_297_MOESM7_ESM.zip › Figure 6A/new 6A PARP.tif]

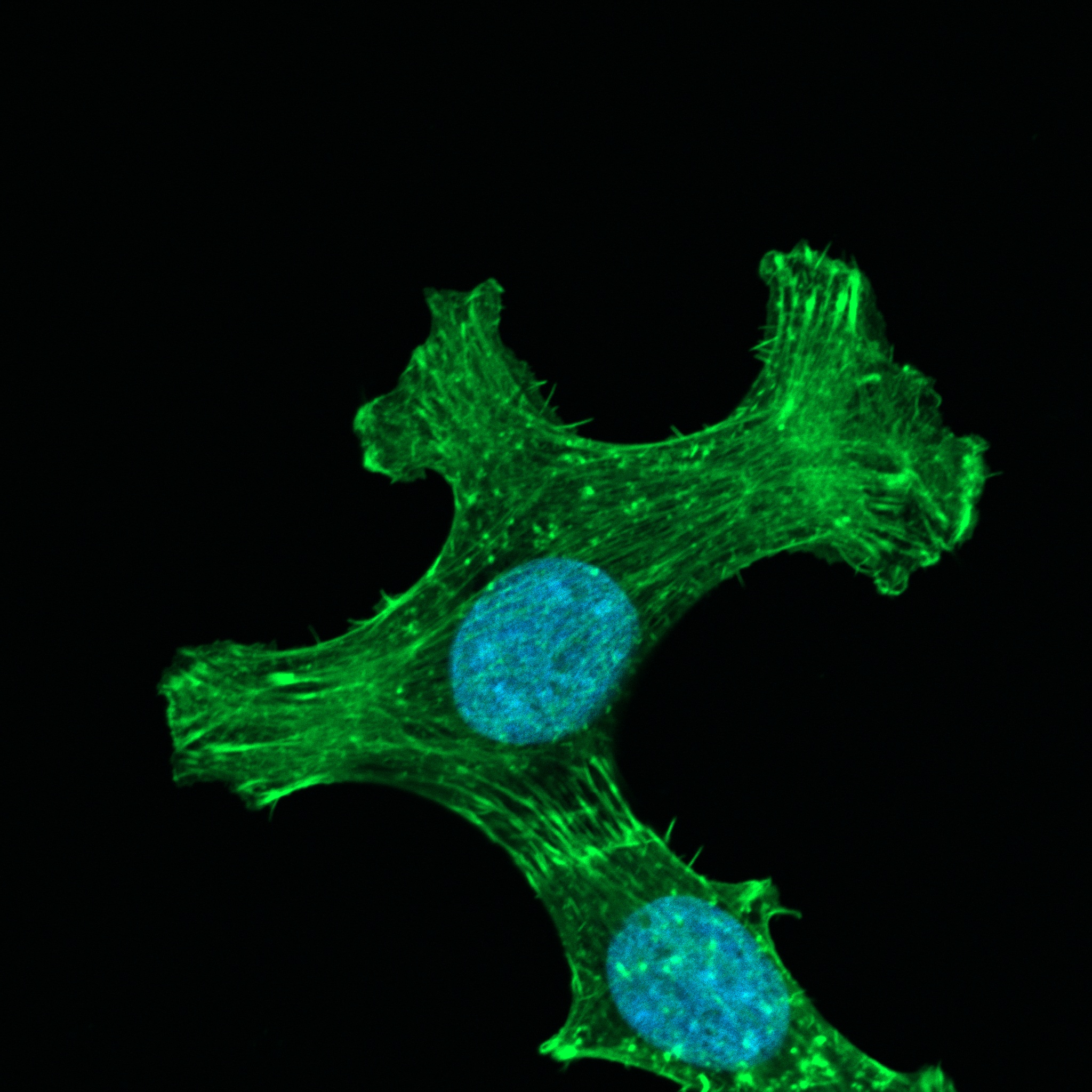

Supplement: Supplementary file 8 — Source data Fig. 7 [file 44321_2025_297_MOESM8_ESM.zip › Figure 7/Figure 7C/A875-shp75 100ngml ngf 63X_h0t0z0c0-2x0-2048y0-2048.jpg]

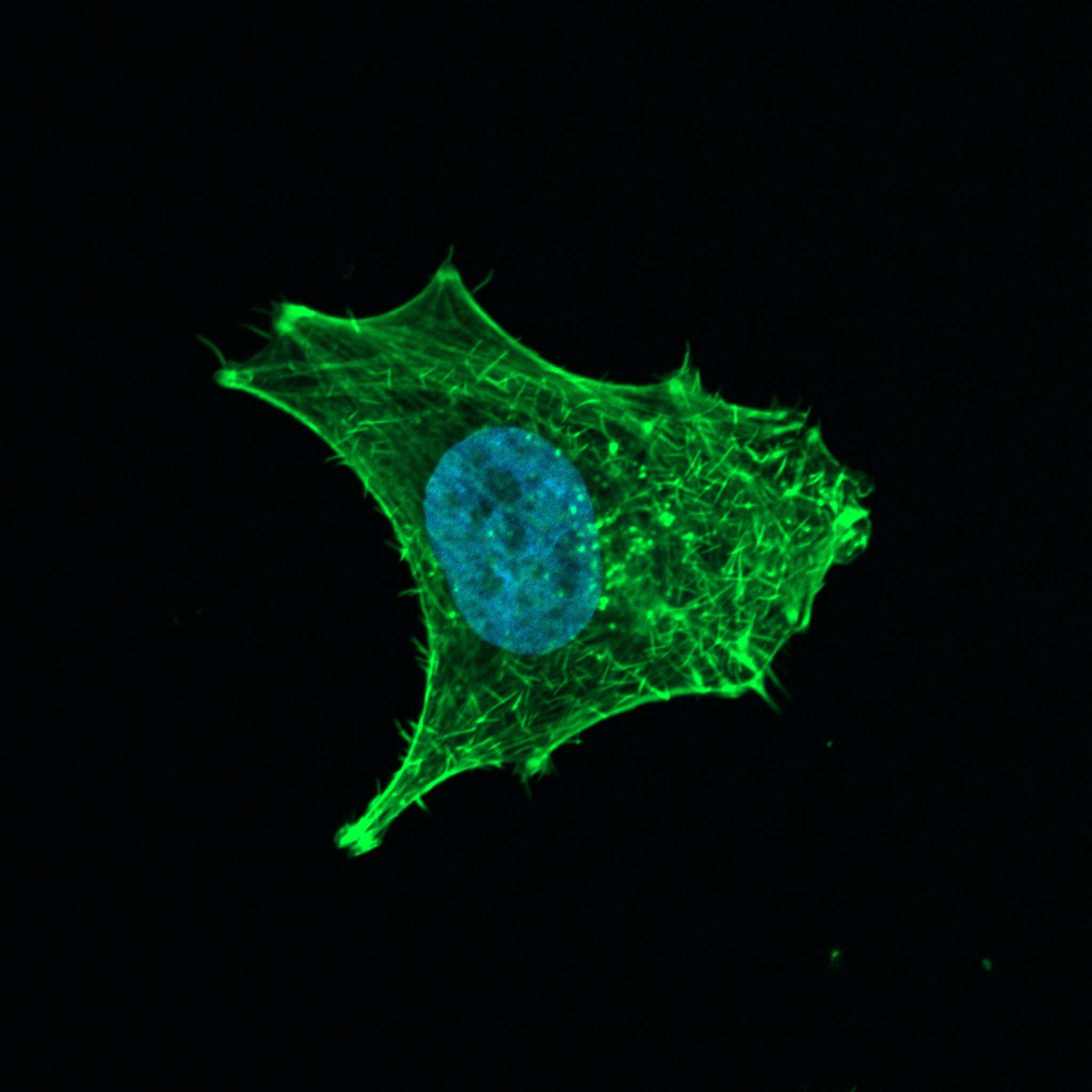

Supplement: Supplementary file 8 — Source data Fig. 7 [file 44321_2025_297_MOESM8_ESM.zip › Figure 7/Figure 7C/A875-nt 100ngml ngf&1um 4a22 63X_h0t0z0c0-2x0-2048y0-2048.jpg]

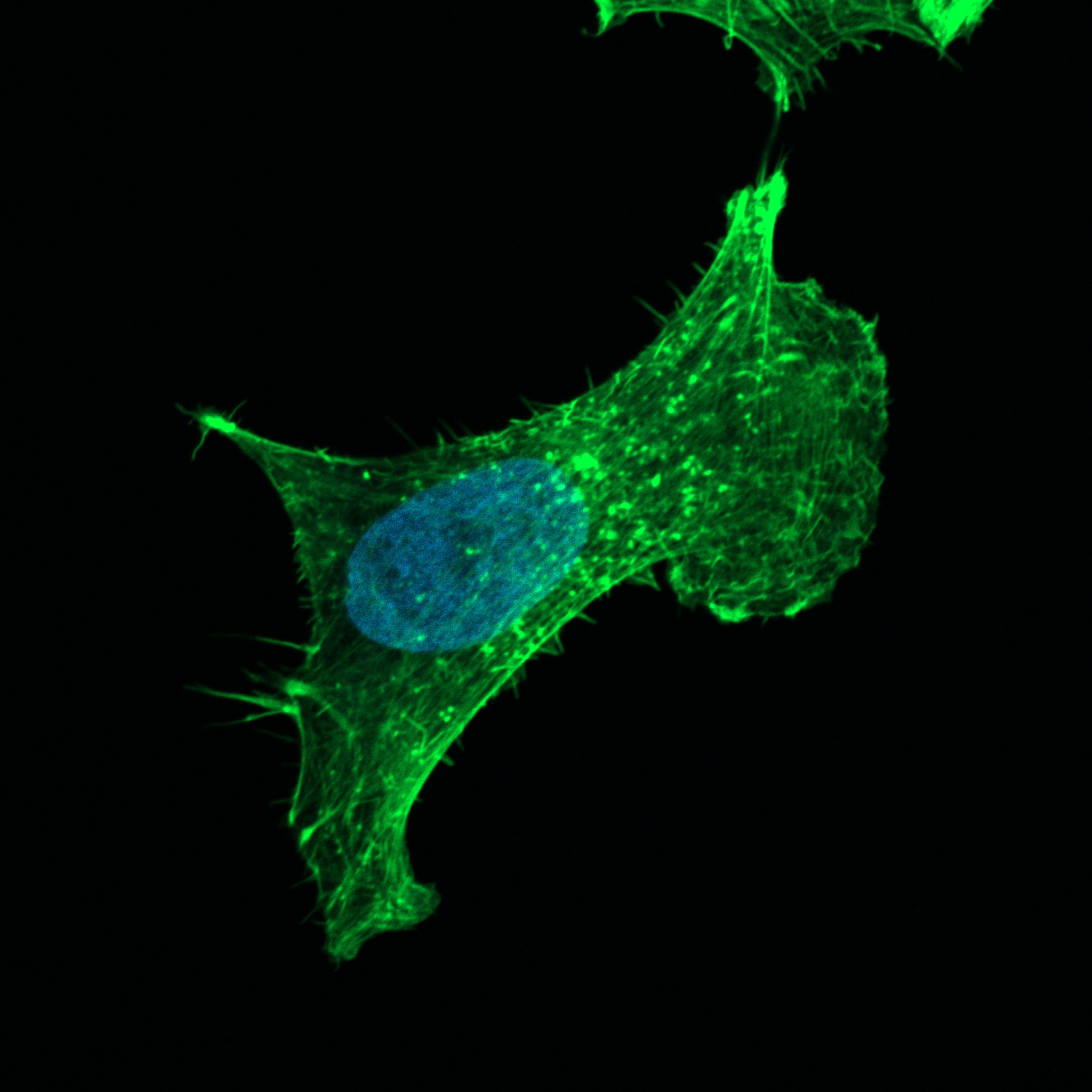

Supplement: Supplementary file 8 — Source data Fig. 7 [file 44321_2025_297_MOESM8_ESM.zip › Figure 7/Figure 7C/A875-shp75 100ngml ngf&5um 4a22 63X_h0t0z0c0-2x0-2048y0-2048.jpg]

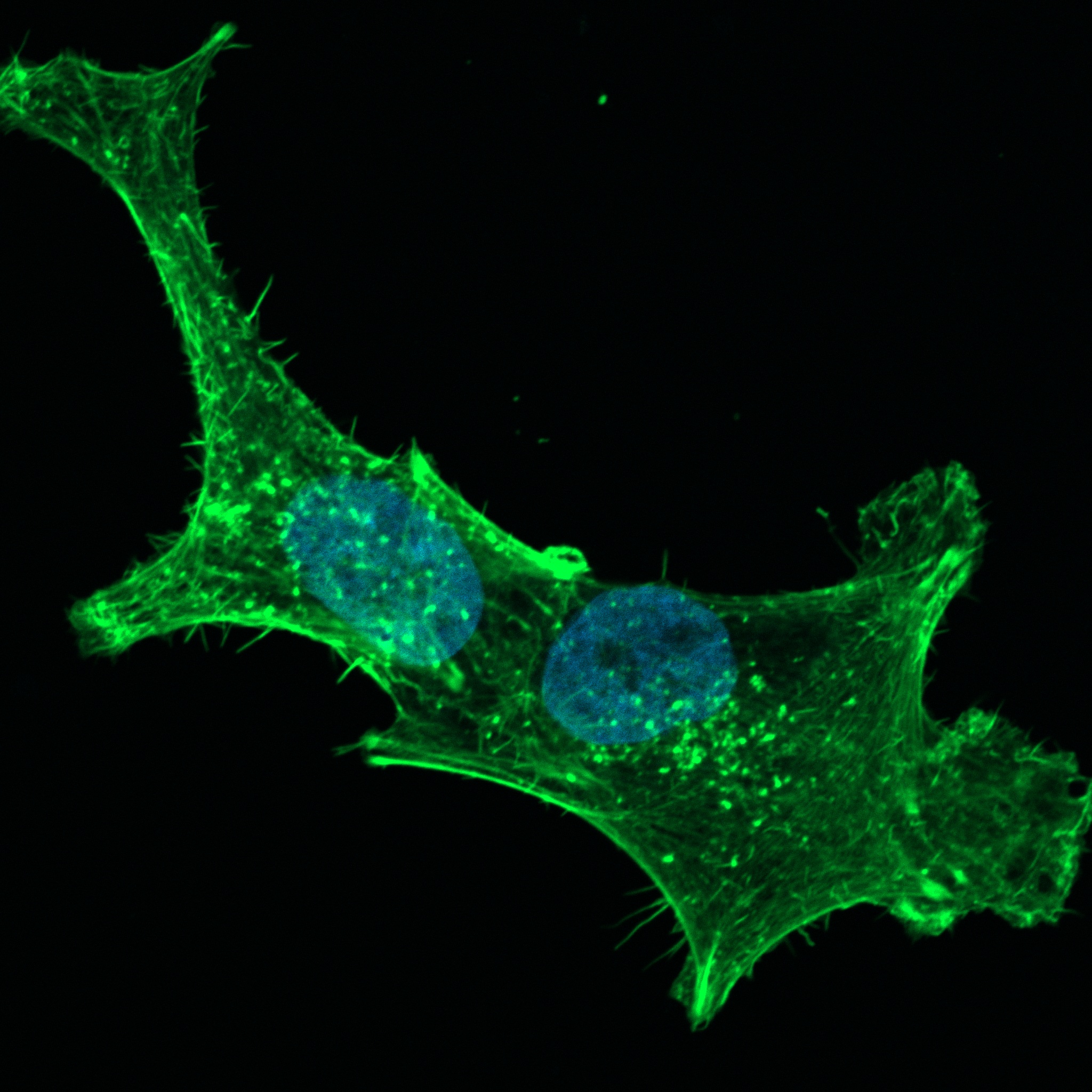

Supplement: Supplementary file 8 — Source data Fig. 7 [file 44321_2025_297_MOESM8_ESM.zip › Figure 7/Figure 7C/A875-shp75 5um 4a22 63X_h0t0z0c0-2x0-2048y0-2048.jpg]

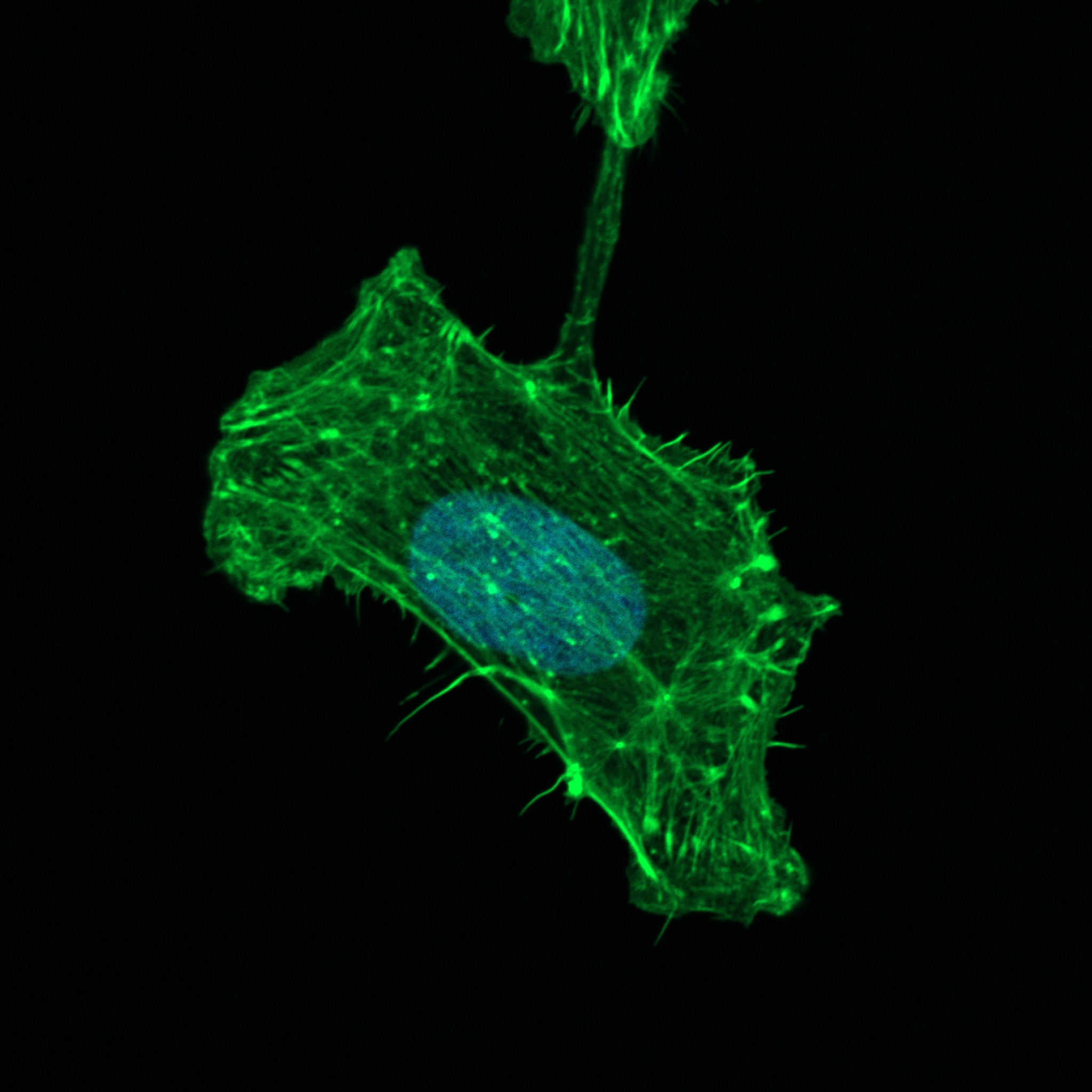

Supplement: Supplementary file 8 — Source data Fig. 7 [file 44321_2025_297_MOESM8_ESM.zip › Figure 7/Figure 7C/A875-shp75 1um 4a22 63X_h0t0z0c0-2x0-2048y0-2048.jpg]

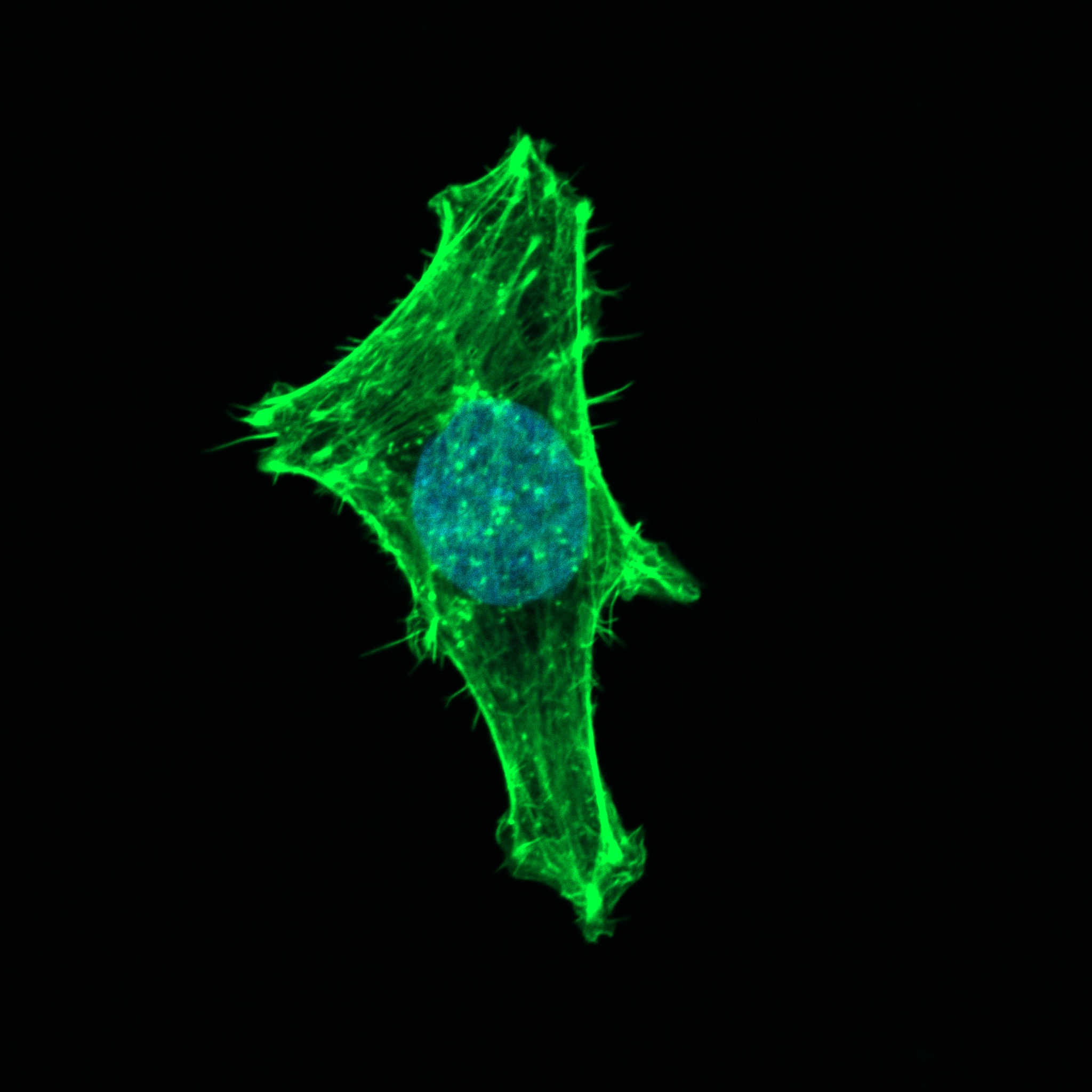

Supplement: Supplementary file 8 — Source data Fig. 7 [file 44321_2025_297_MOESM8_ESM.zip › Figure 7/Figure 7C/A875-shp75 DMSO 63X_h0t0z0c0-2x0-2048y0-2048.jpg]

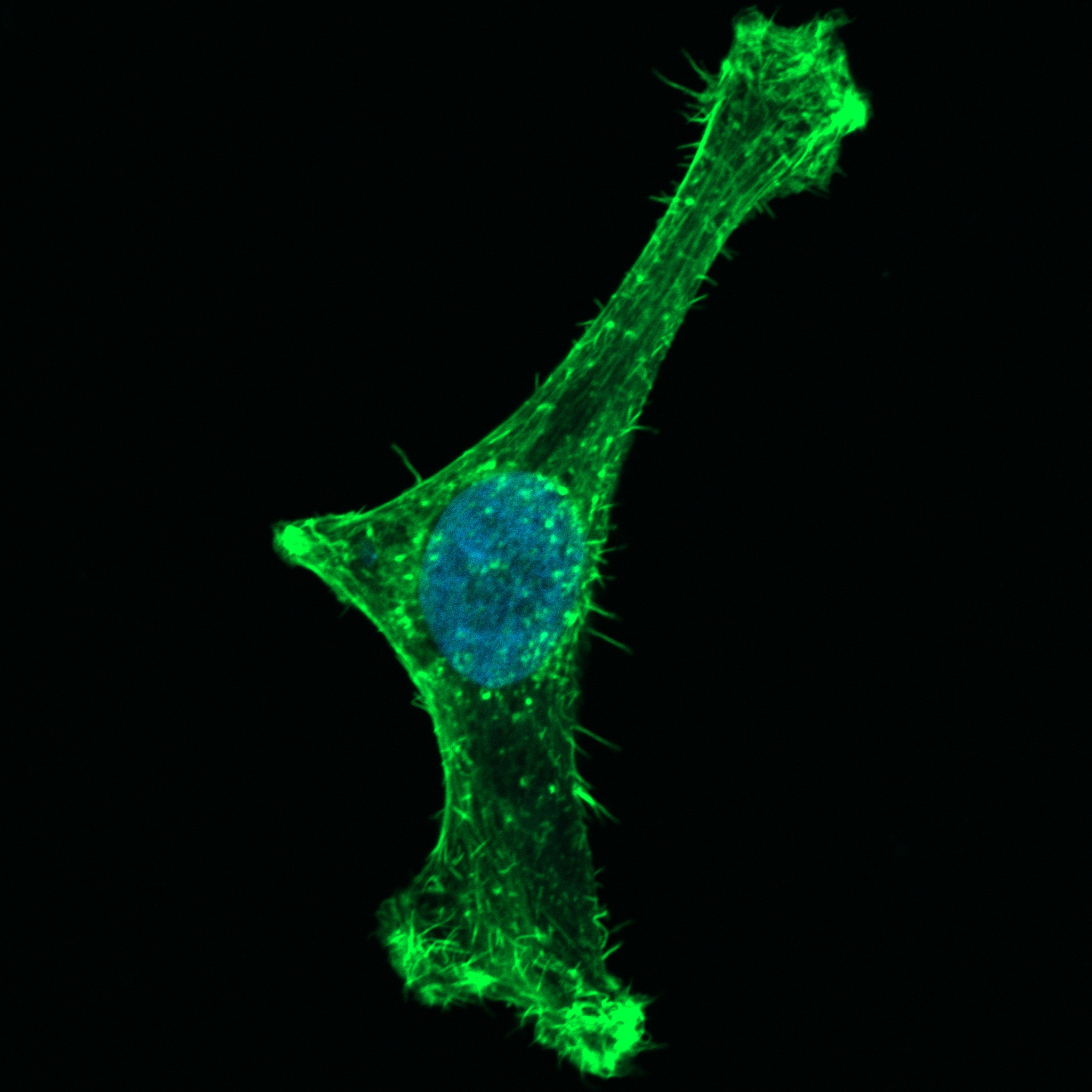

Supplement: Supplementary file 8 — Source data Fig. 7 [file 44321_2025_297_MOESM8_ESM.zip › Figure 7/Figure 7C/A875-shp75 100ngml ngf&1um 4a22 63X_h0t0z0c0-2x0-2048y0-2048.jpg]

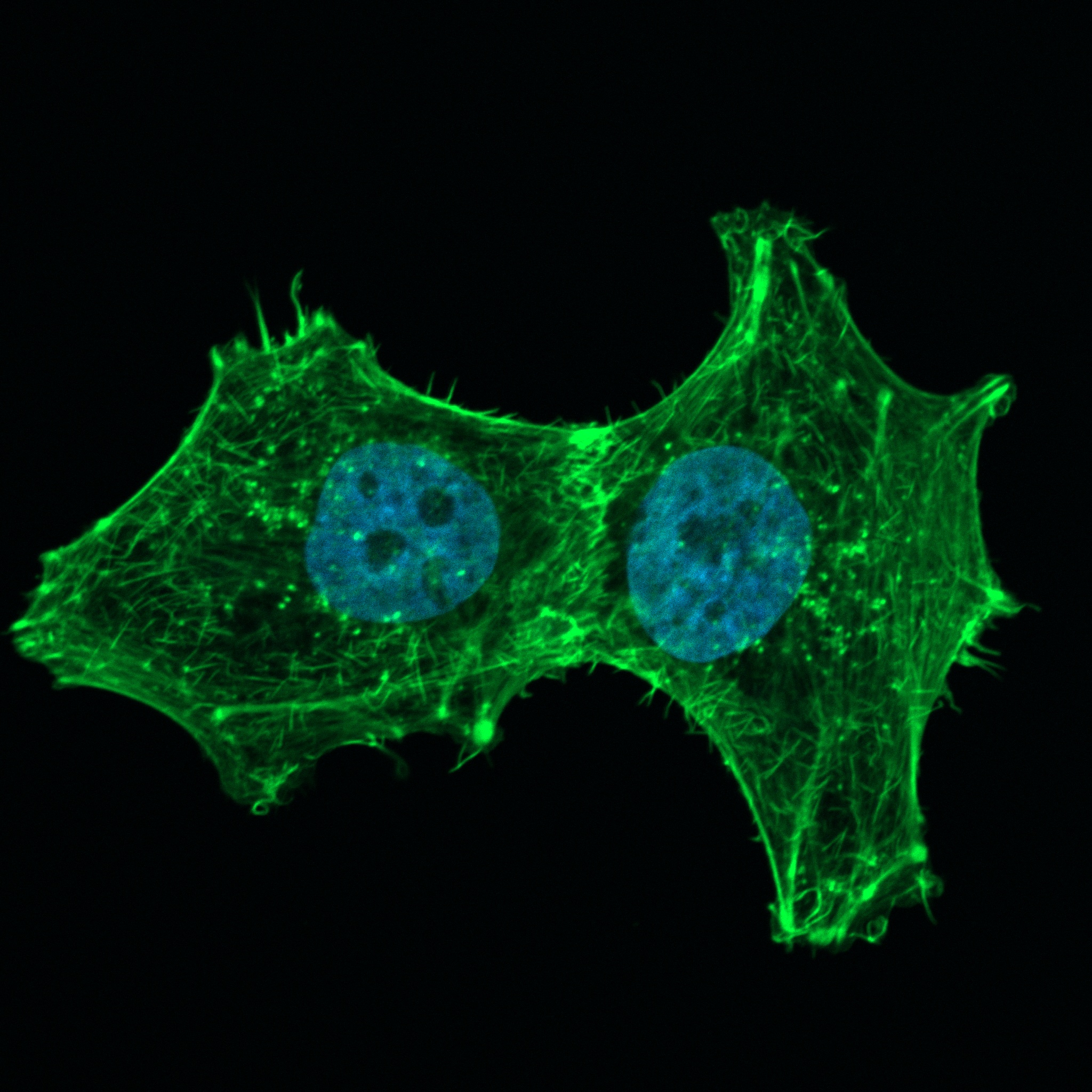

Supplement: Supplementary file 8 — Source data Fig. 7 [file 44321_2025_297_MOESM8_ESM.zip › Figure 7/Figure 7C/A875-nt 100ngml ngf&5um 4a22 63X_h0t0z0c0-2x0-2048y0-2048.jpg]

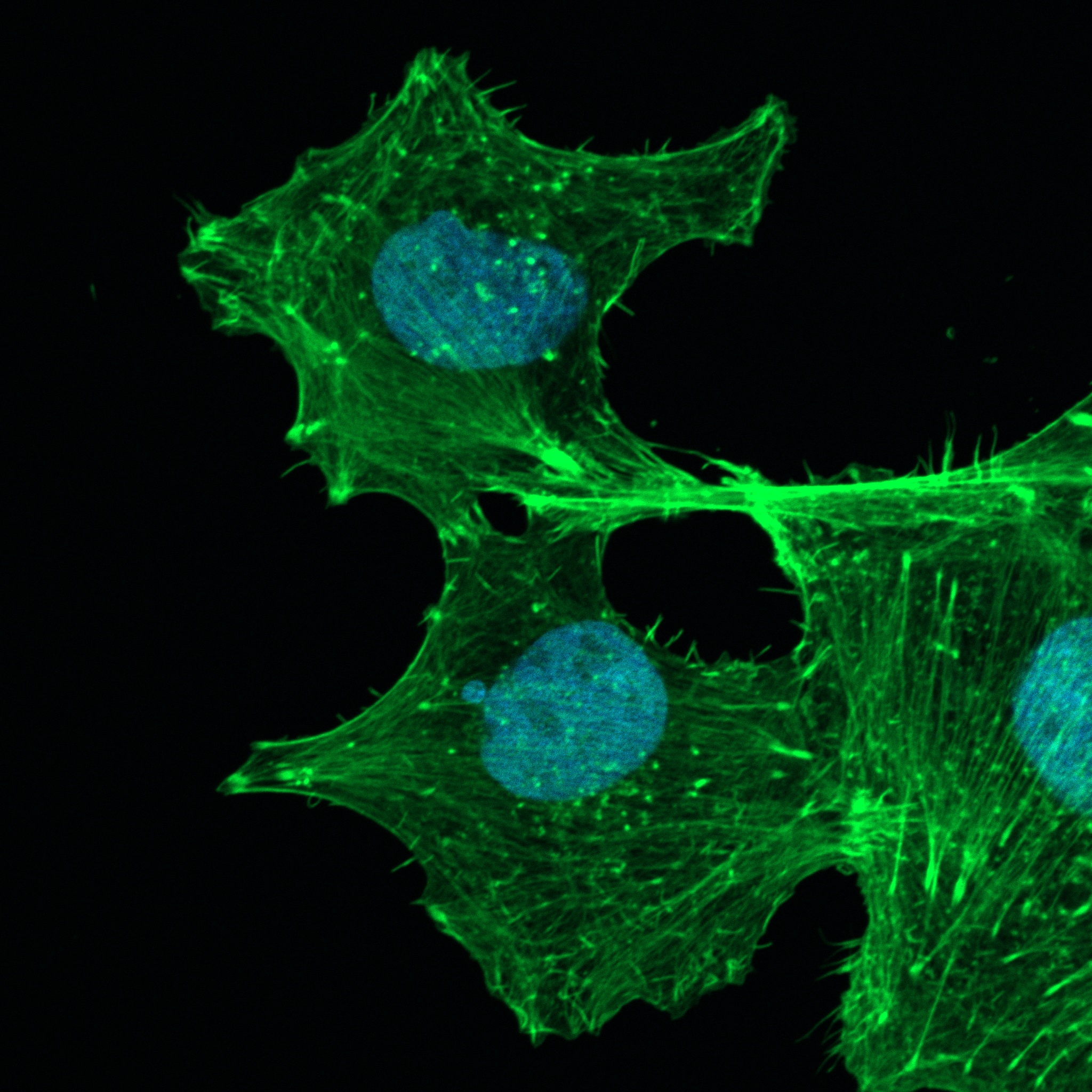

Supplement: Supplementary file 8 — Source data Fig. 7 [file 44321_2025_297_MOESM8_ESM.zip › Figure 7/Figure 7C/A875-nt 5um 4a22 63X_h0t0z0c0-2x0-2048y0-2048.jpg]

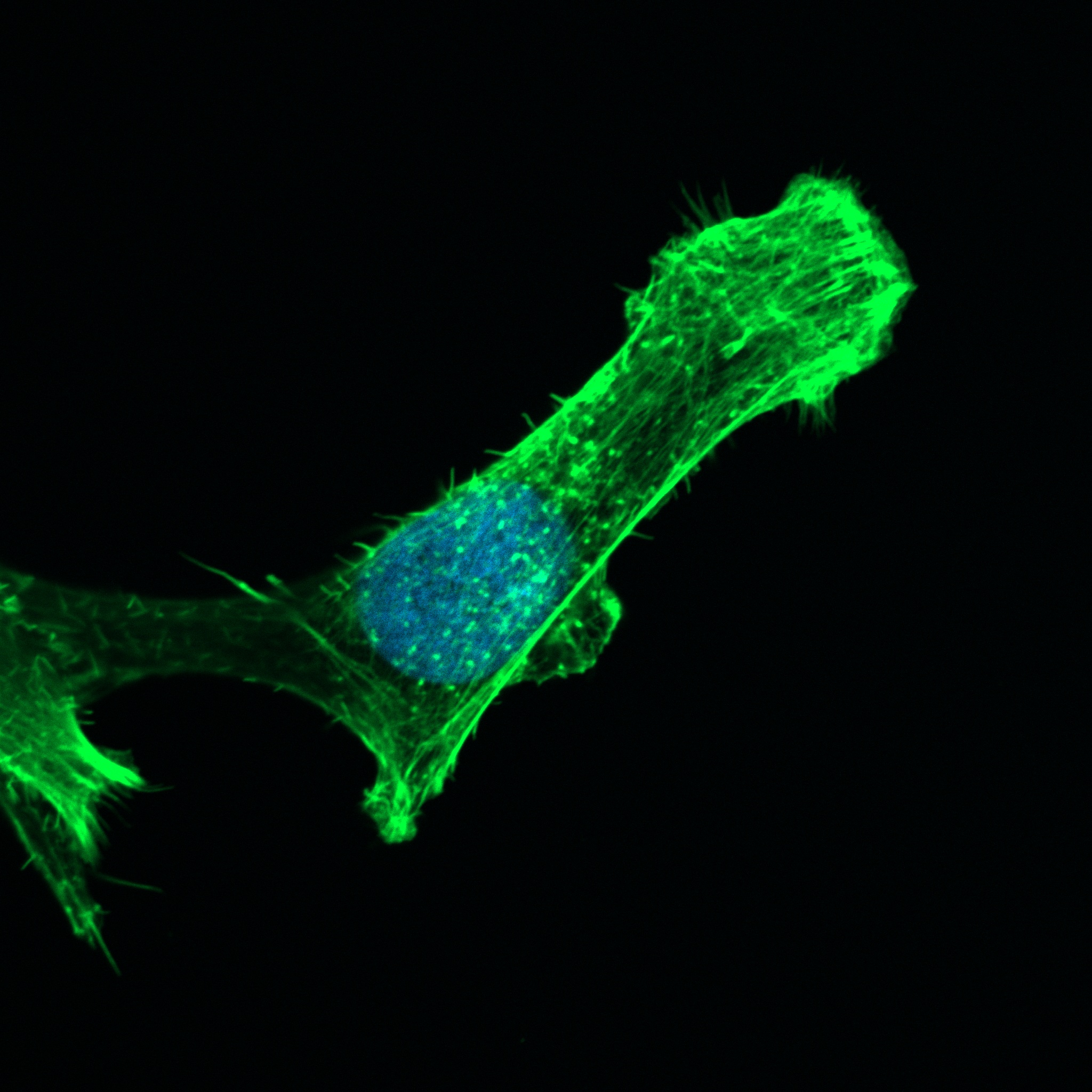

Supplement: Supplementary file 8 — Source data Fig. 7 [file 44321_2025_297_MOESM8_ESM.zip › Figure 7/Figure 7C/A875-nt dmso 63X_h0t0z0c0-2x0-2048y0-2048.jpg]

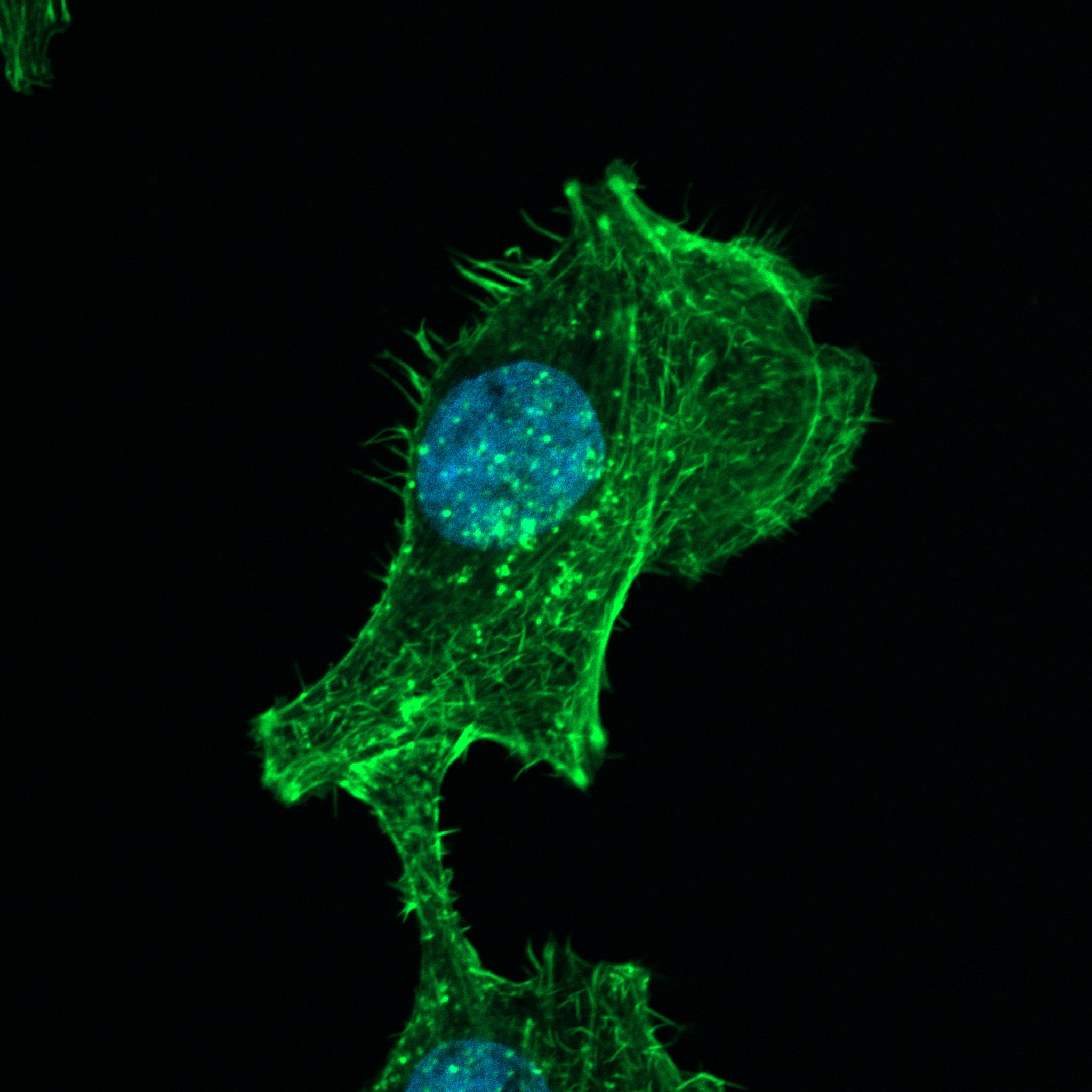

Supplement: Supplementary file 8 — Source data Fig. 7 [file 44321_2025_297_MOESM8_ESM.zip › Figure 7/Figure 7C/A875-nt 100ngml ngf 63X_h0t0z0c0-2x0-2048y0-2048.jpg]

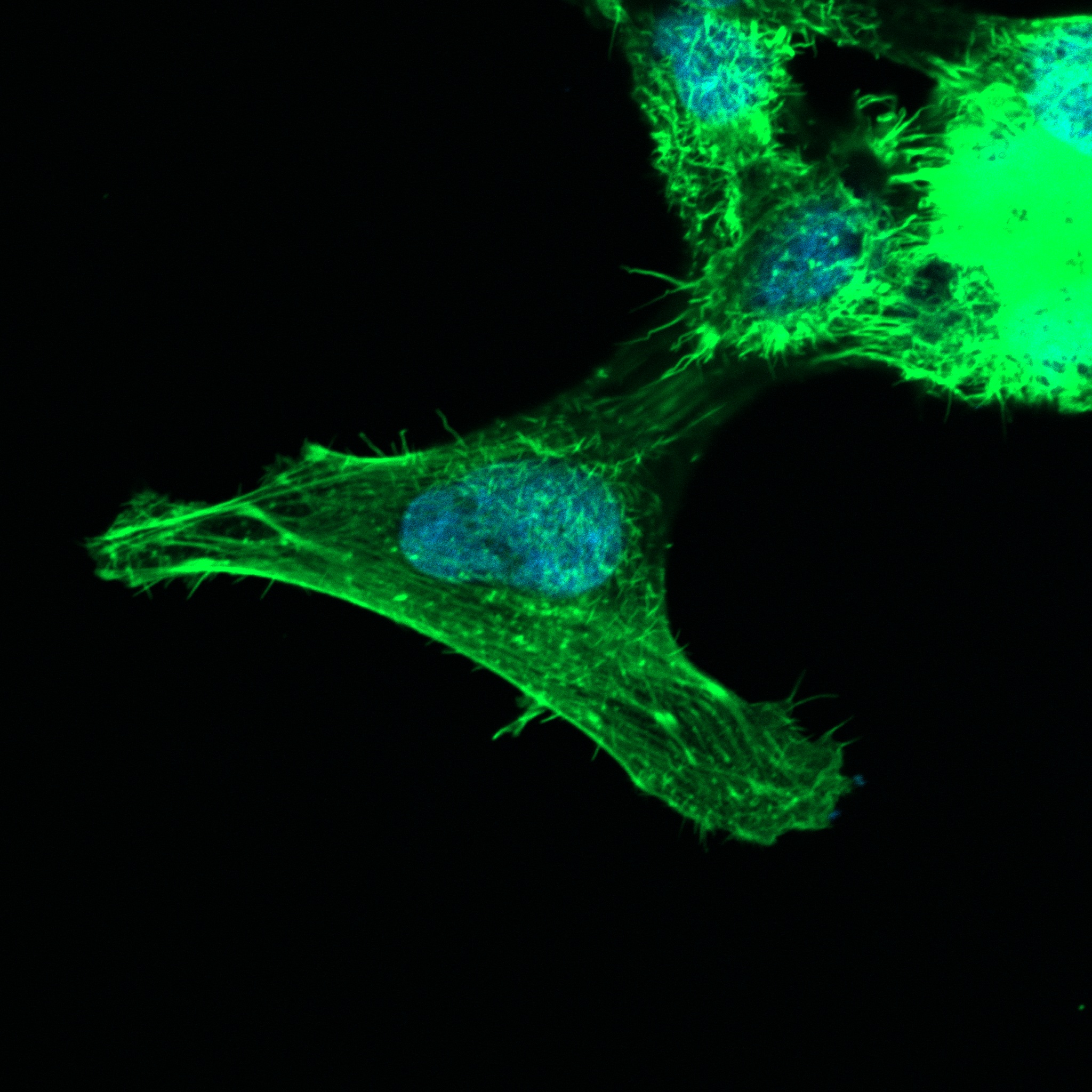

Supplement: Supplementary file 8 — Source data Fig. 7 [file 44321_2025_297_MOESM8_ESM.zip › Figure 7/Figure 7C/A875-nt 1um 4a22 63X_h0t0z0c0-2x0-2048y0-2048.jpg]

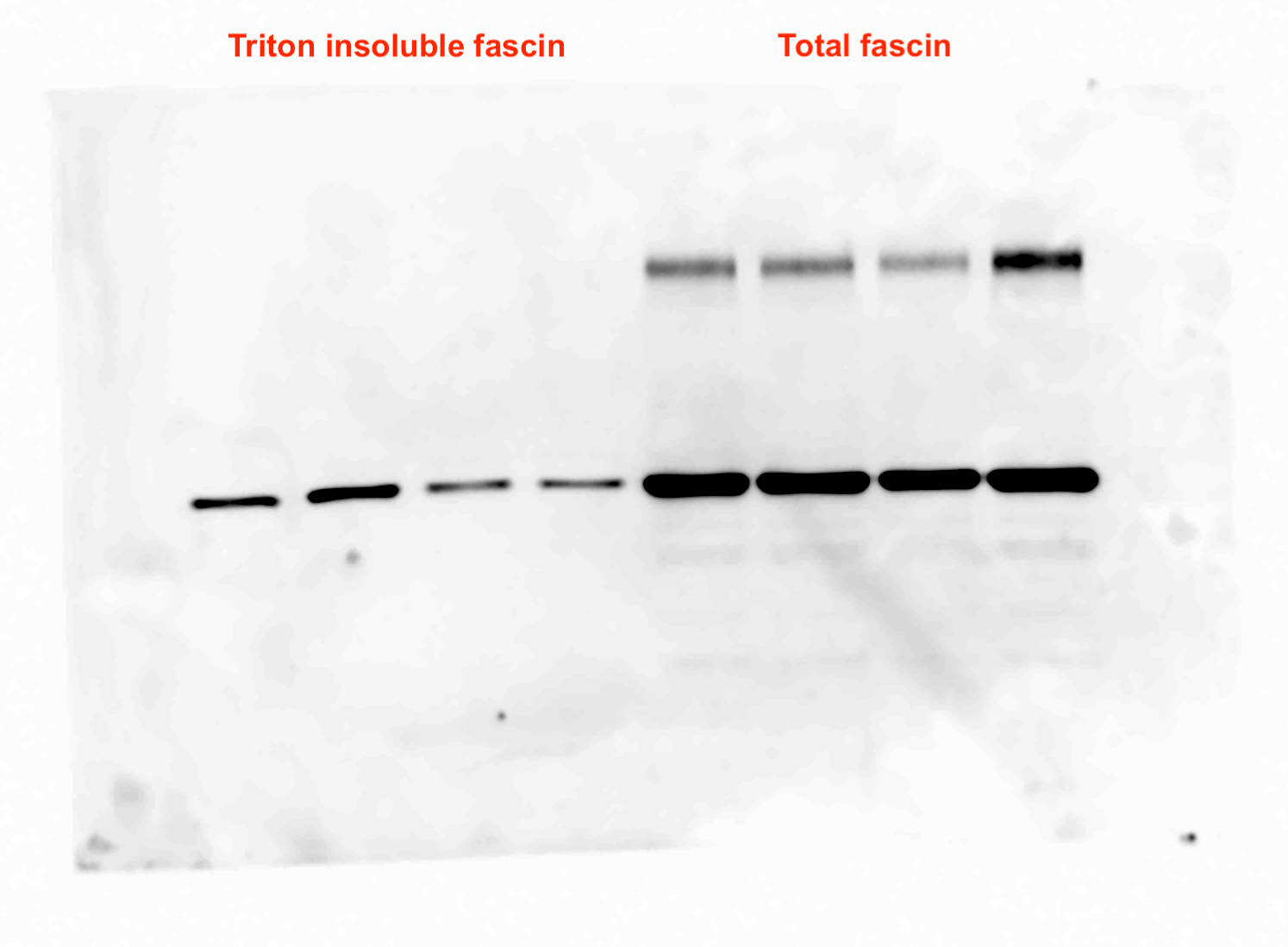

Supplement: Supplementary file 8 — Source data Fig. 7 [file 44321_2025_297_MOESM8_ESM.zip › Figure 7/Figure 7B/Figure 7B.tif]

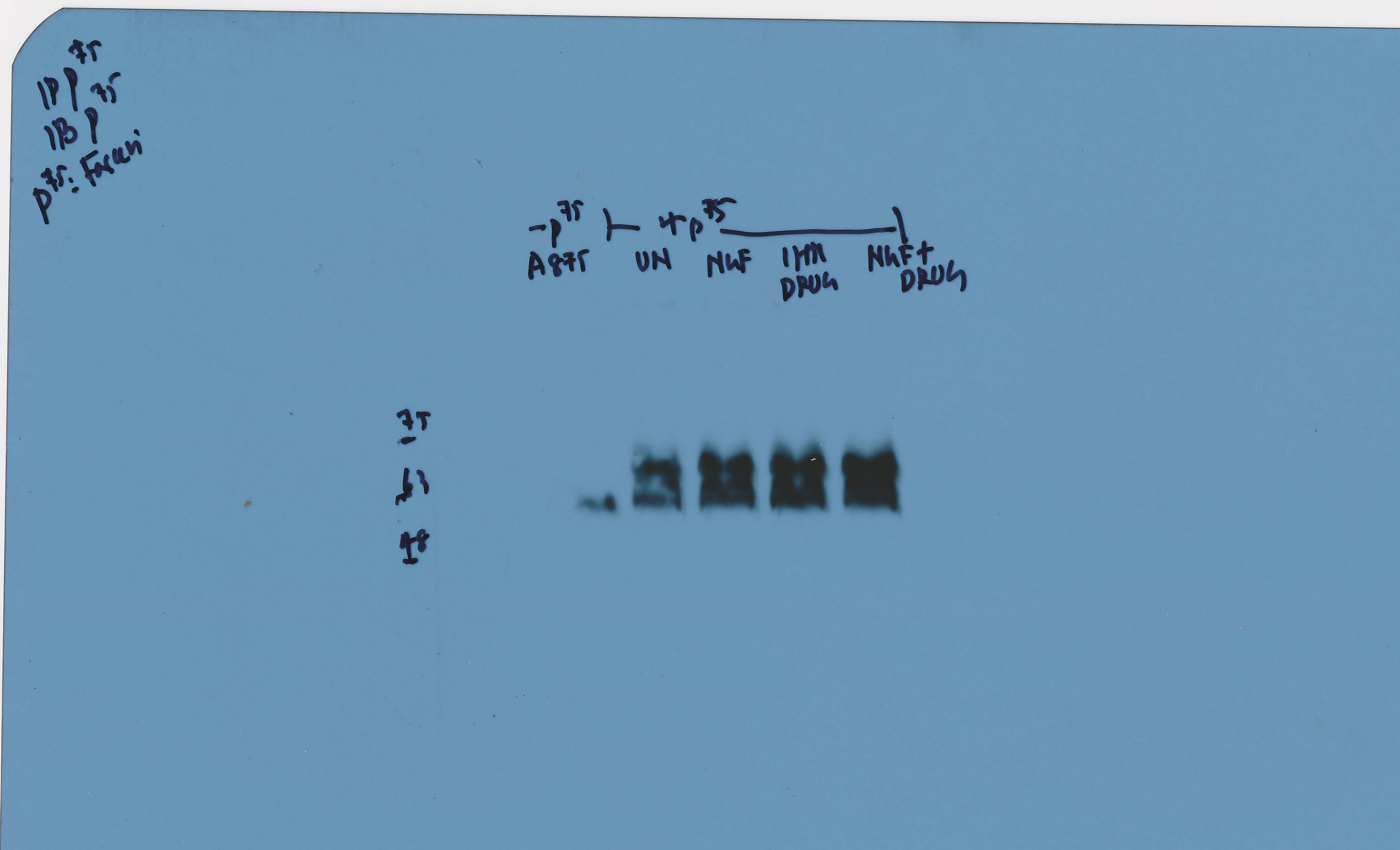

Supplement: Supplementary file 8 — Source data Fig. 7 [file 44321_2025_297_MOESM8_ESM.zip › Figure 7/Figure 7A/IP p75NTR IB p75NTR.jpg]

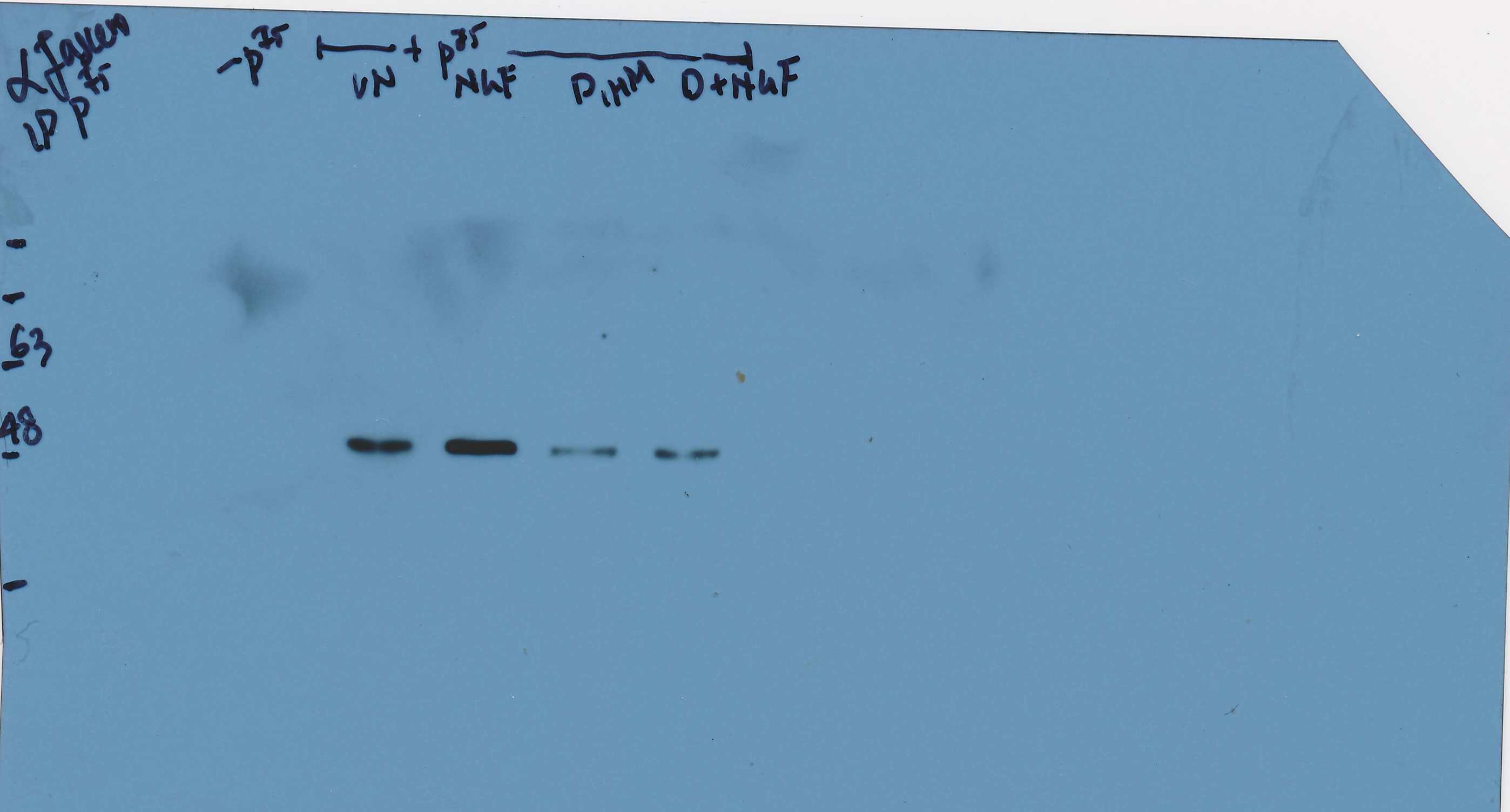

Supplement: Supplementary file 8 — Source data Fig. 7 [file 44321_2025_297_MOESM8_ESM.zip › Figure 7/Figure 7A/IP p75NTR IB Fascin.jpg]

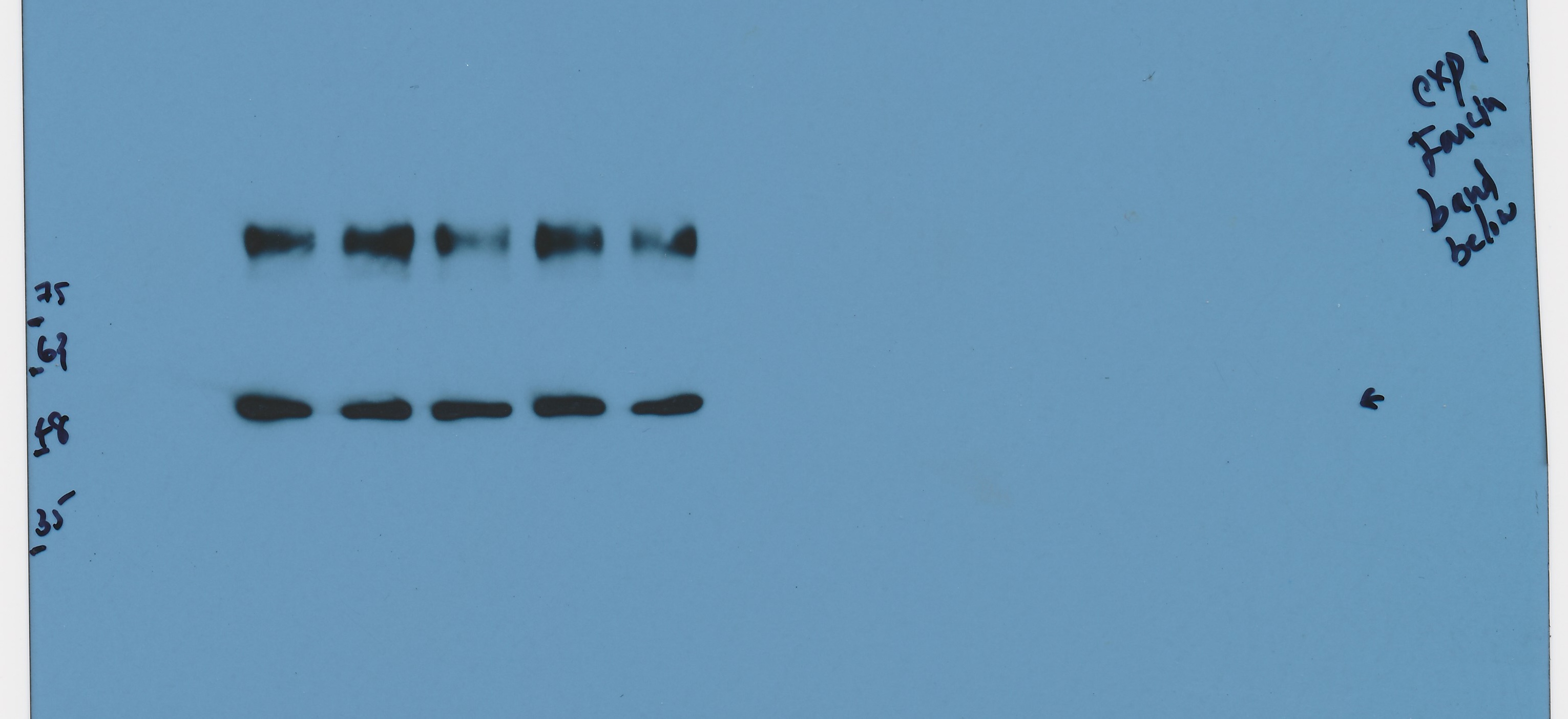

Supplement: Supplementary file 8 — Source data Fig. 7 [file 44321_2025_297_MOESM8_ESM.zip › Figure 7/Figure 7A/WCL Fascin.jpg]

## Slide 1
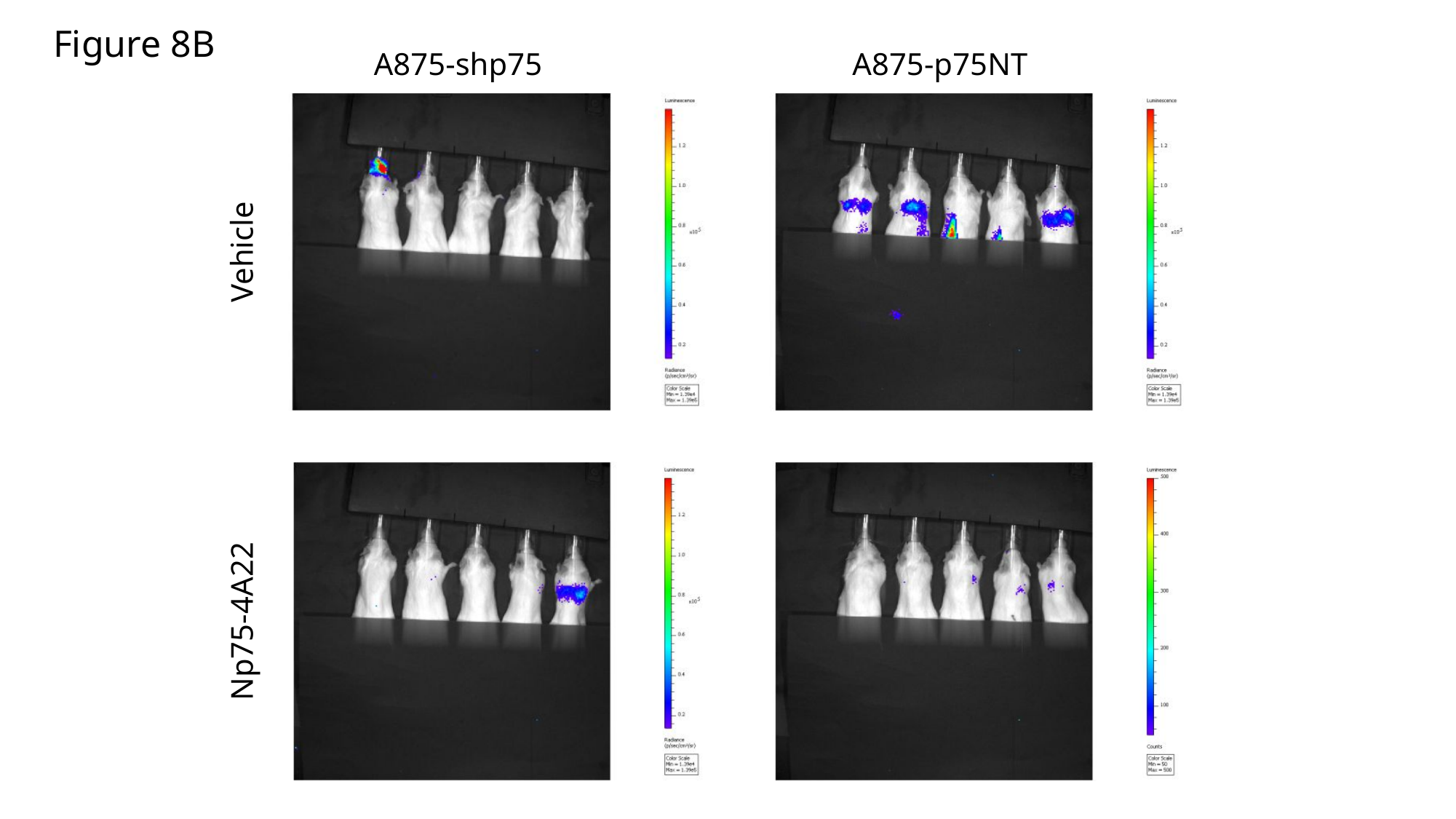

Figure 8B
A875-shp75
A875-p75NT
Vehicle
Np75-4A22

Supplement: Supplementary file 9 — Source data Fig. 8 [file 44321_2025_297_MOESM9_ESM.zip › Figure 8/Figure 8B.pptx]

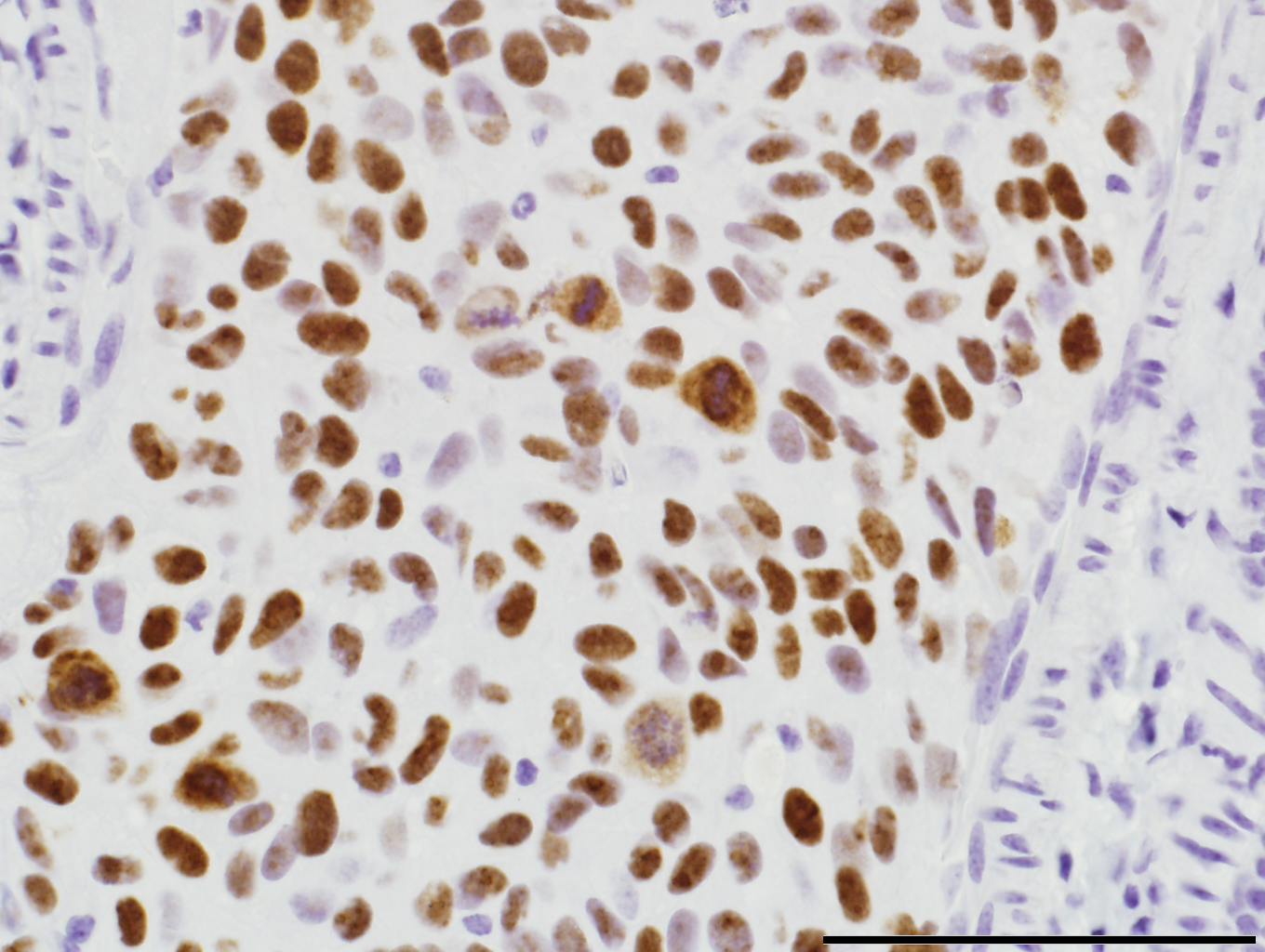

Supplement: Supplementary file 9 — Source data Fig. 8 [file 44321_2025_297_MOESM9_ESM.zip › Figure 8/Figure 8C/A875 NT Vehicle Control 60x- Fig 8C.tif]

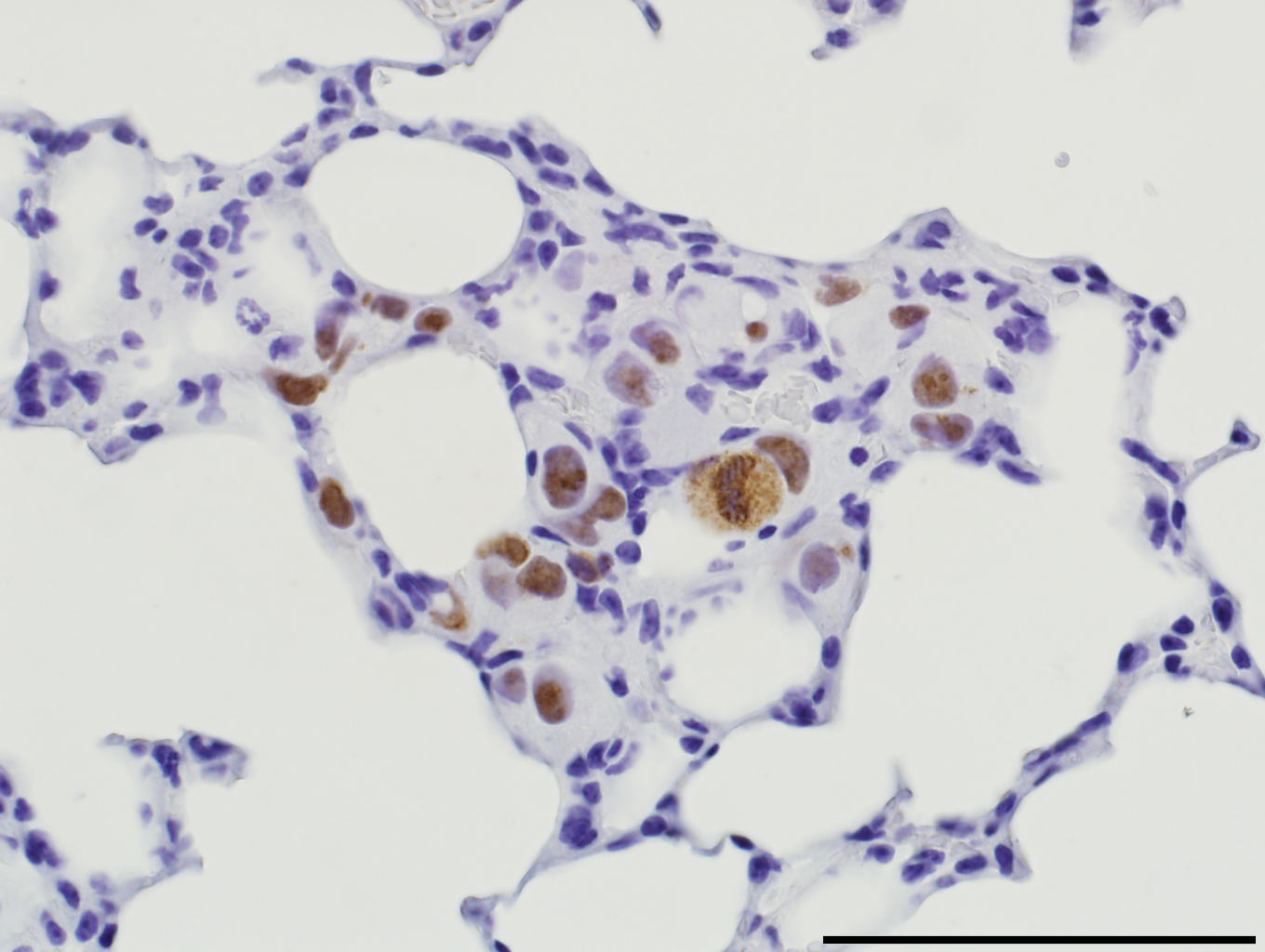

Supplement: Supplementary file 9 — Source data Fig. 8 [file 44321_2025_297_MOESM9_ESM.zip › Figure 8/Figure 8C/A875 shp75 4A22 60X- Fig 8C 2.tif]

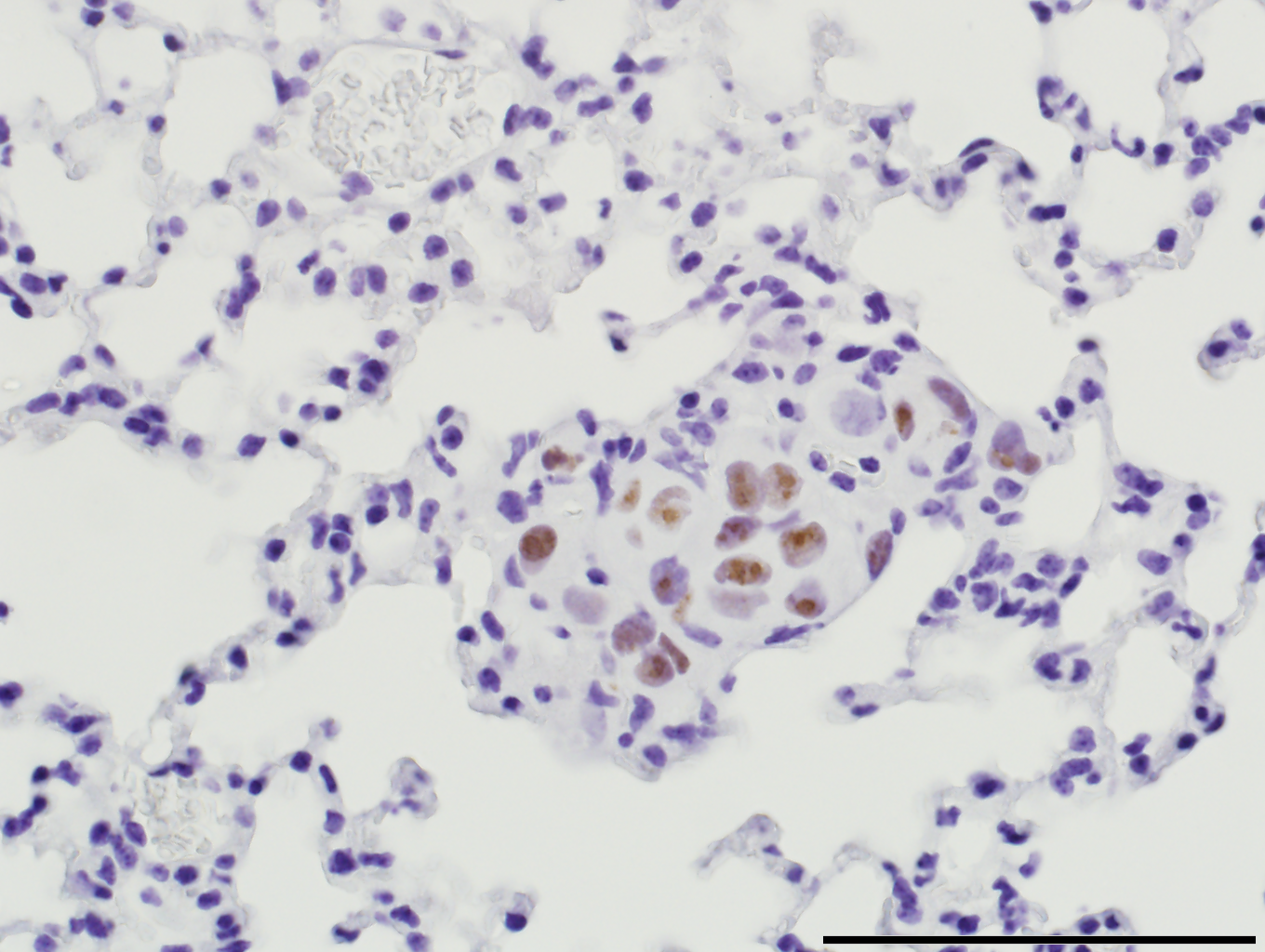

Supplement: Supplementary file 9 — Source data Fig. 8 [file 44321_2025_297_MOESM9_ESM.zip › Figure 8/Figure 8C/A875 NT 4A22 60x- Fig 8C.tif]
