# Supplementary material for: Impaired migration and lung invasion of human melanoma by a novel small molecule targeting the transmembrane domain of death receptor p75NTR
Source: EMBO Mol Med. 2025 Sep 17;17(10):2661–90. doi: 10.1038/s44321-025-00297-1 (PMC12514245; doi:10.1038/s44321-025-00297-1)
Supplement: Supplementary file 10 — Expanded View Figures [file 44321_2025_297_MOESM10_ESM.pdf]

Expanded View Figures

Series 1/2= 41 compounds

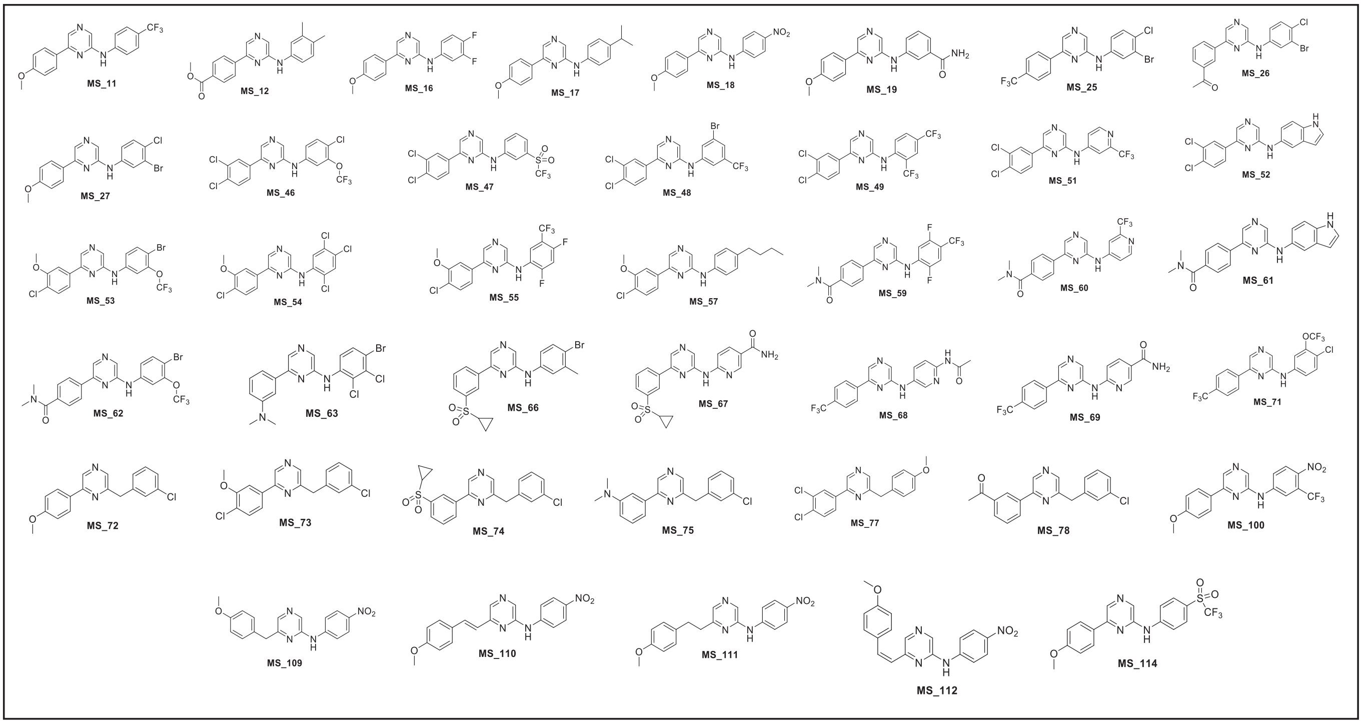

Figure EV1. Chemical structures of Div17E5 analogs: Series 1 and 2.

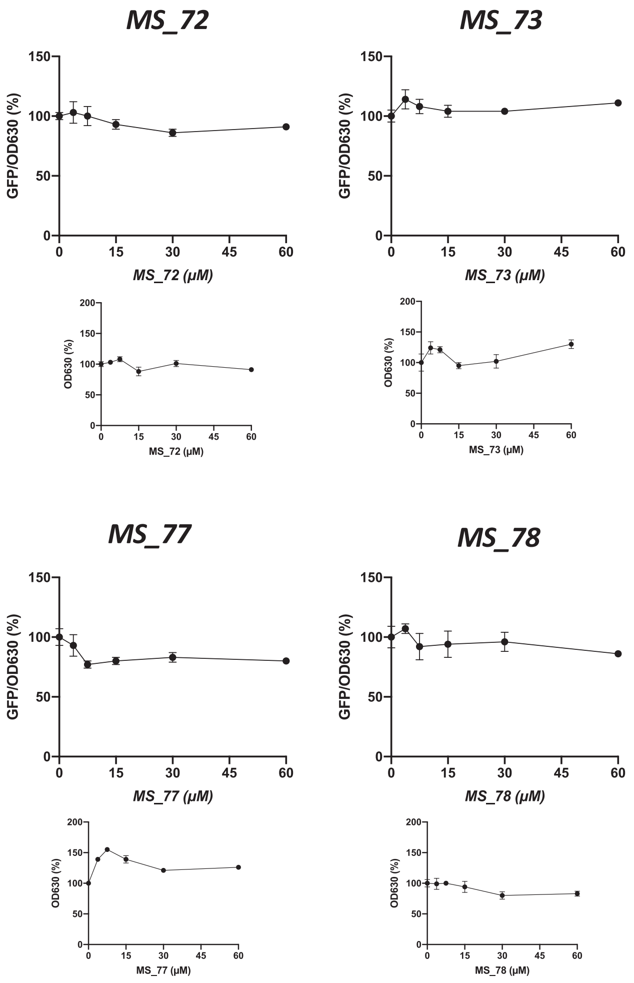

Figure EV2. GFP/OD630 and OD630 of compounds in Series 2A.

 © The Author(s)

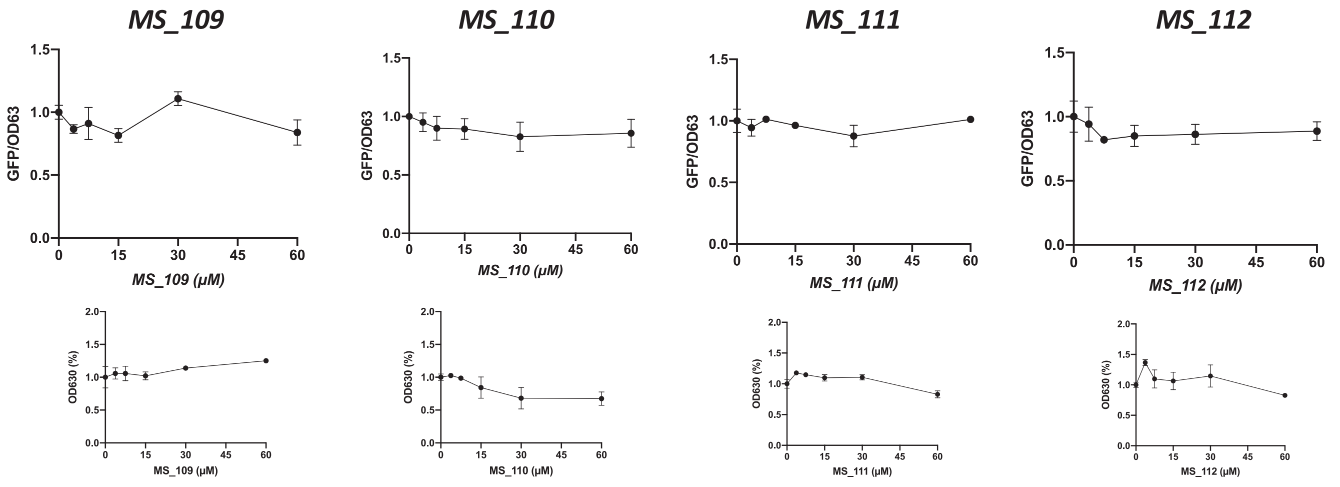

Figure EV3. GFP/OD630 and OD630 of compounds in Series 2B.

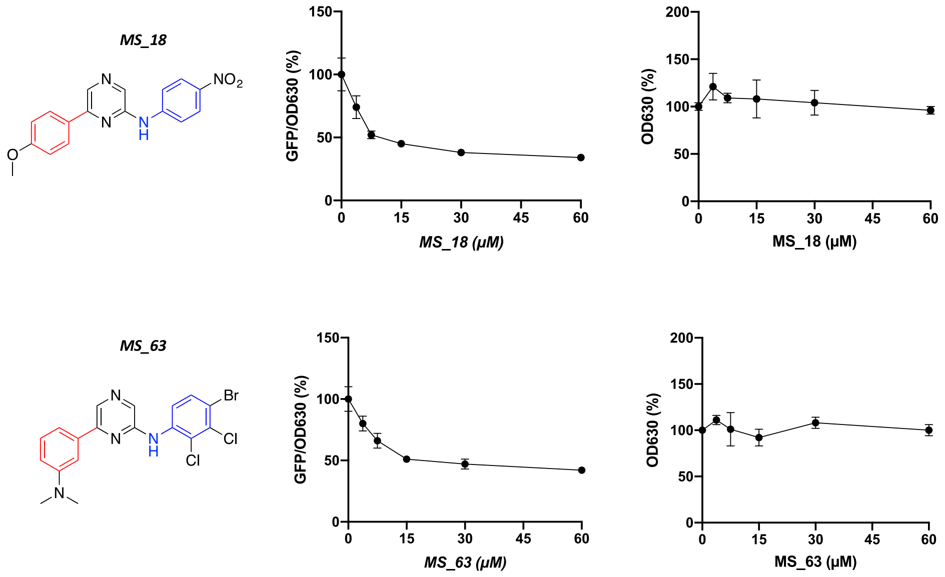

Figure EV4. GFP/OD630 and OD630 of compounds MS_18 and MS_63.

 © The Author(s)

3A Series = 51 compounds

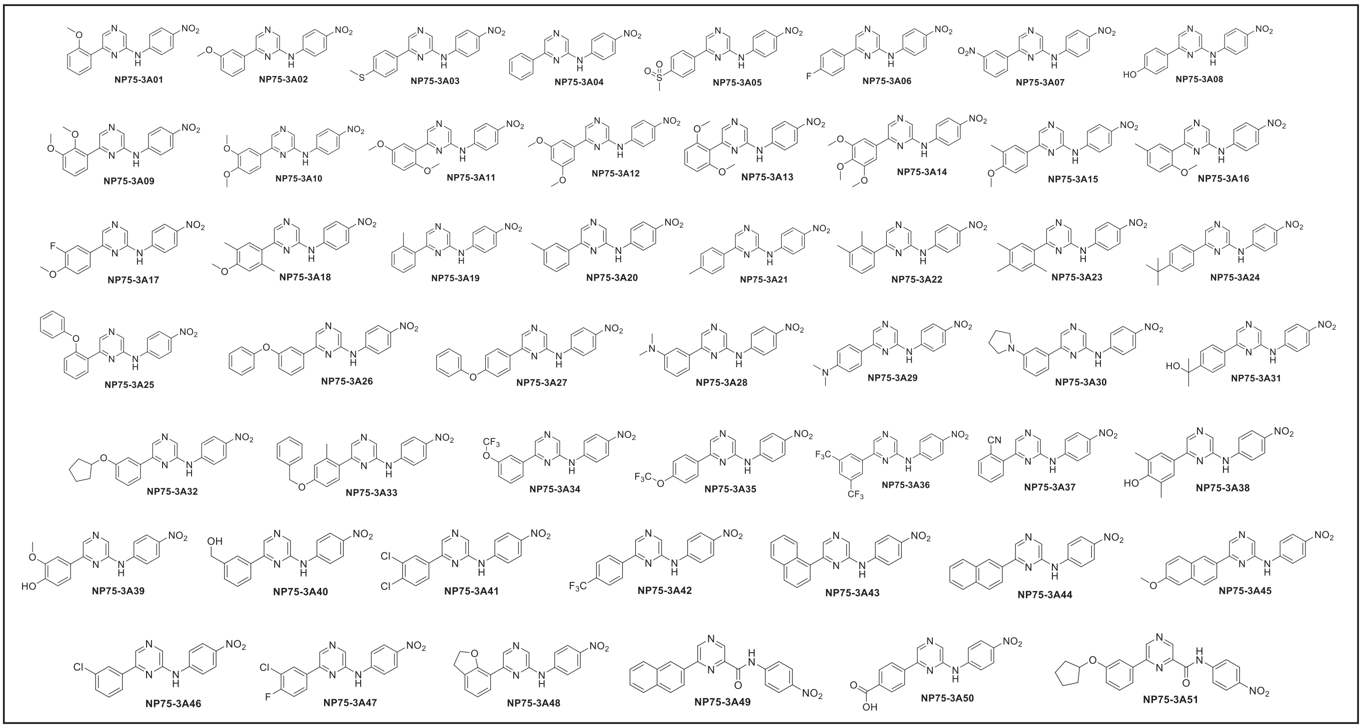

Figure EV5. Chemical structures of Div17E5 analogs: Series 3A.

3B Series = 41 compounds

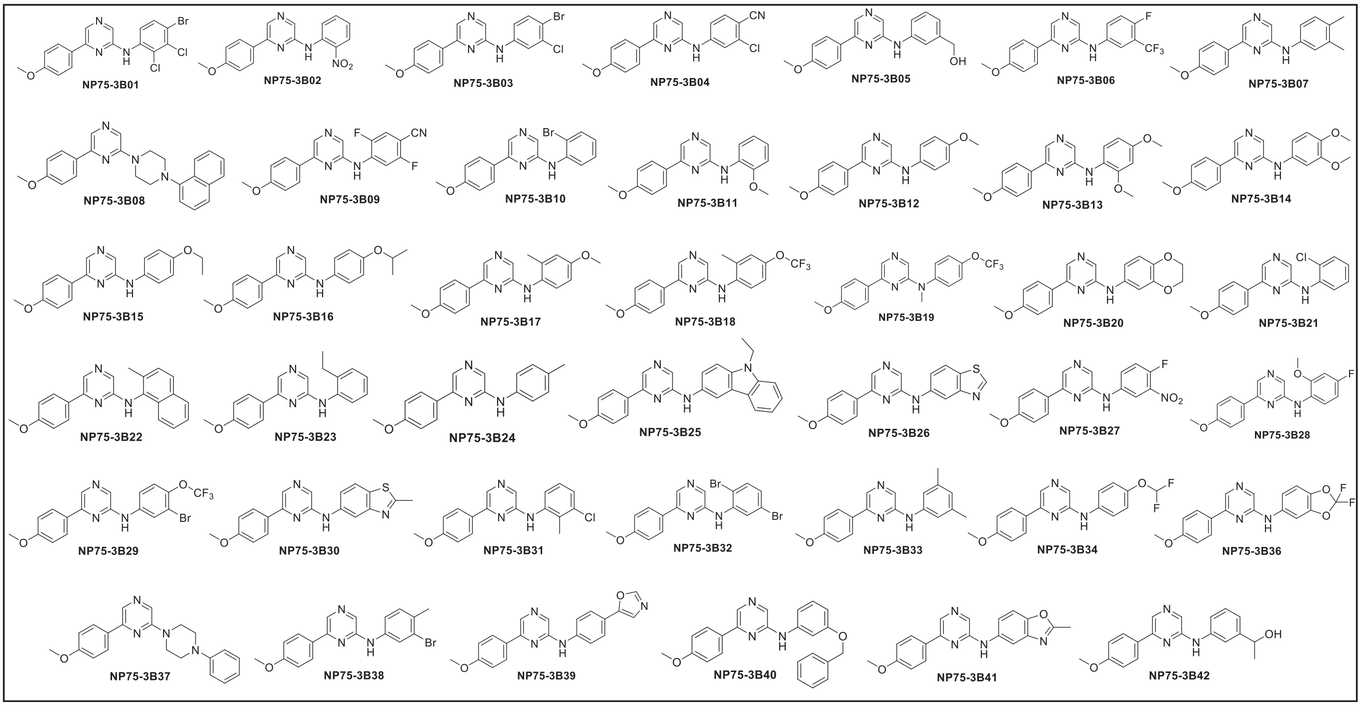

Figure EV6. Chemical structures of Div17E5 analogs: Series 3B.

 © The Author(s)

4A Series = 27 compounds

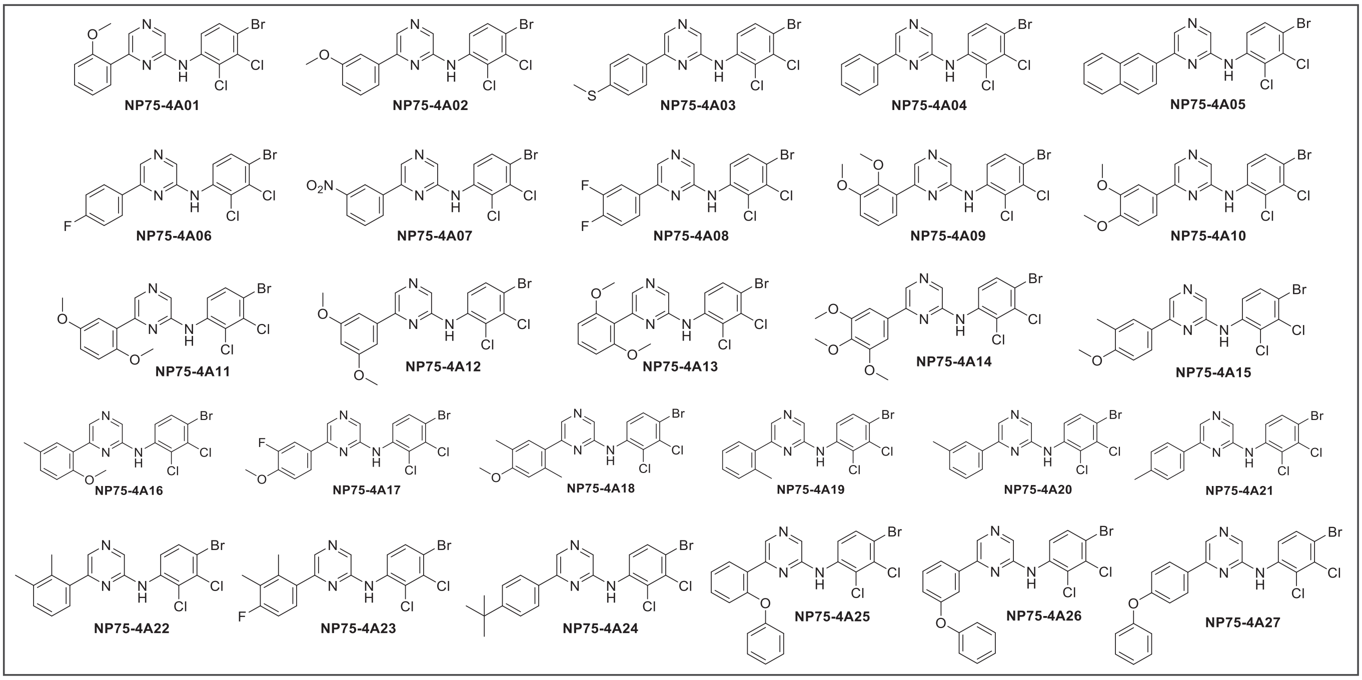

Figure EV7. Chemical structures of Div17E5 analogs: Series 4A.

4B Series = 25 compounds

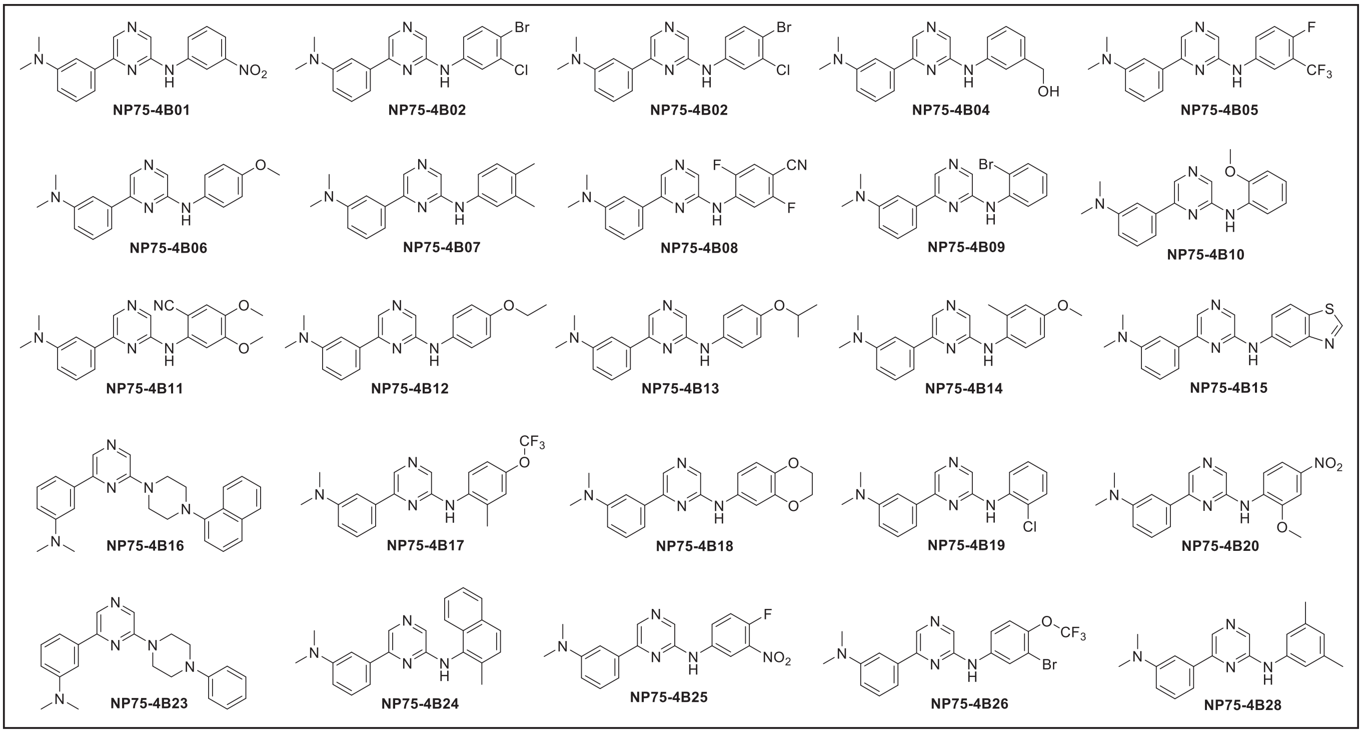

Figure EV8. Chemical structures of Div17E5 analogs: Series 4B.

A

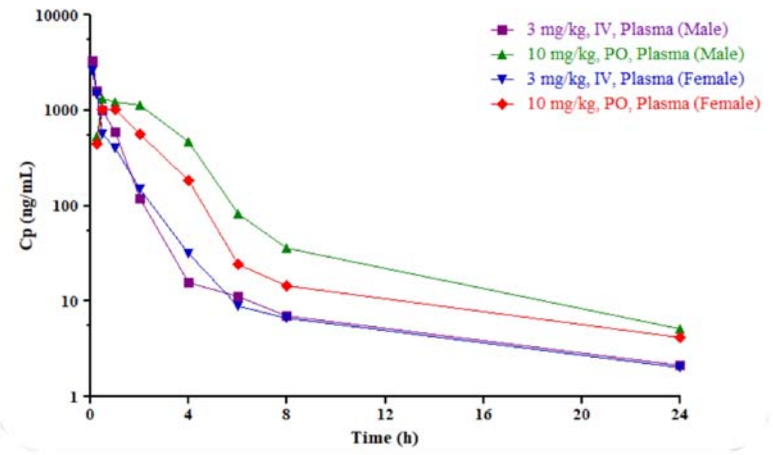

B

Matrix	Route	Gender	Dose (mg/kg)	T_{max} (h)	C_0/C_{max} (ng/mL)	AUC_{last} (h*ng/mL)	$T_{1/2}$ (h)	CL (mL/min/kg)	V_{ss} (L/kg)	%F
Plasma	IV	Male	3	-	4714.86	2069.50	7.45	23.91	2.09	-
		Female	3	-	3398.84	1670.78	8.64	29.49	3.23	-
	PO	Male	10	0.50	1314.61	4728.68	-	-	-	**69**
		Female	10	0.50	1026.67	2683.00	-	-	-	**48**

a – Back extrapolated concentration in IV group.

Figure EV9. Pharmacokinetic study of Np75-4A22 in mice.

(**A**) Graph shows plasma concentrations of Np75-4A22 in male and female C57BL/6 mice ($N = 9$) after a single intravenous (3 mg/kg) or oral (10 mg/kg) administration of 10 mg/kg. Plasma was collected at pre-dose, 0.25, 0.5, 1, 2, 4, 5, 10 and 24 hs and assessed for Np75-4A22 using LC/MS/MS. (**B**) Pharmacokinetic parameters of Np75-4A22 in plasma following a single intravenous (3 mg/kg) or oral (10 mg/kg) in male and female C57BL/6 mice. Cmax: maximum (peak) plasma drug concentration (amount/volume). Tmax: time to reach maximum (peak) plasma concentration following drug administration. AUClast: area under the plasma concentration-time curve from time zero to time of last measurable concentration (time/volume). Bioavailability (%F): extent and rate at which the active moiety (drug or metabolite) enters systemic circulation.

© The Author(s)

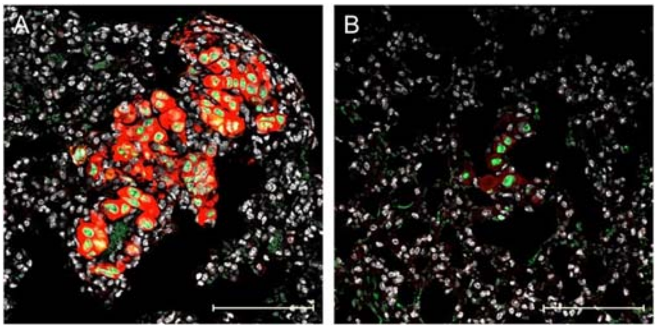

Figure EV10. Histological analysis of p75NTR expression in lung metastasis produced by A875 cells.

$p75^{NTR}$ immunostaining (red) in lung metastasis induced by A875-NT (**A**) or shp75-A875 (**B**) cells counter-stained for human nucleolin (green) and DAPI (white). Scale bar, 100 μM.

 © The Author(s)
